# Pathogenic Interleukin-10 Receptor Alpha Variants in Humans — Balancing Natural Selection and Clinical Implications

**DOI:** 10.1007/s10875-022-01366-7

**Published:** 2022-11-12

**Authors:** Dominik Aschenbrenner, Ziqing Ye, Ying Zhou, Wenhui Hu, Isabel Brooks, Isabelle Williams, Melania Capitani, Lisa Gartner, Daniel Kotlarz, Scott B. Snapper, Christoph Klein, Aleixo M. Muise, Brian D. Marsden, Ying Huang, Holm H. Uhlig

**Affiliations:** 1 Translational Gastroenterology Unit, Experimental Medicine, John Radcliffe Hospital, University of Oxford, Oxford, OX3 9DU UK; 2 Present Address: Novartis Institutes for BioMedical Research, Novartis Pharma AG, Basel, Switzerland; 3 Department of Gastroenterology, National Children’s Medical Center, Children’s Hospital of Fudan University, 399 Wanyuan Road, Shanghai, 201102 China; 4 Present Address: SenTcell Ltd., London, UK; 5 Dr. von Hauner Children’s Hospital, Department of Pediatrics, University Hospital, Ludwig-Maximilians-Universität Munich, Munich, Germany; 6 Institute of Translational Genomics, Helmholtz Zentrum München - German Research Center for Environmental Health, Neuherberg, Germany; 7 Boston Children’s Hospital and Harvard Medical School, Boston, MA 02115 USA; 8 Gene Center, LMU Munich, Munich, Germany; 9 Deutsche Zentrum für Infektionsforschung (DZIF) and Deutsches Zentrum für Kinder- und Jugendgesundheit, Partner site Munich, Munich, Germany; 10 SickKids Inflammatory Bowel Disease Centre and Cell Biology Program, Research Institute, The Hospital for Sick Children, Toronto, Canada; 11 Division of Gastroenterology, Hepatology, and Nutrition, Department of Pediatrics, Toronto, Canada; 12 The Hospital for Sick Children, University of Toronto, Toronto, ON Canada; 13 Centre of Medicines Discovery, NDM, University of Oxford, Oxford, OX3 7DQ UK; 14 Kennedy Institute of Rheumatology, NDORMS, University of Oxford, Oxford, OX3 7FY UK; 15 Department of Pediatrics, University of Oxford, Oxford, UK; 16 Biomedical Research Center, University of Oxford, Oxford, UK

**Keywords:** IL-10, *IL10RA*, natural selection, inflammatory bowel disease

## Abstract

Balancing natural selection is a process by which genetic variants arise in populations that are beneficial to heterozygous carriers, but pathogenic when homozygous. We systematically investigated the prevalence, structural, and functional consequences of pathogenic *IL10RA* variants that are associated with monogenic inflammatory bowel disease. We identify 36 non-synonymous and non-sense variants in the *IL10RA* gene. Since the majority of these *IL10RA* variants have not been functionally characterized, we performed a systematic screening of their impact on STAT3 phosphorylation upon IL-10 stimulation. Based on the geographic accumulation of confirmed pathogenic *IL10RA* variants in East Asia and in Northeast China, the distribution of infectious disorders worldwide, and the functional evidence of IL-10 signaling in the pathogenesis, we identify *Schistosoma japonicum* infection as plausible selection pressure driving variation in *IL10RA*. Consistent with this is a partially augmented IL-10 response in peripheral blood mononuclear cells from heterozygous variant carriers. A parasite-driven heterozygote advantage through reduced IL-10 signaling has implications for health care utilization in regions with high allele frequencies and potentially indicates pathogen eradication strategies that target IL-10 signaling.

**Graphical abstract:**

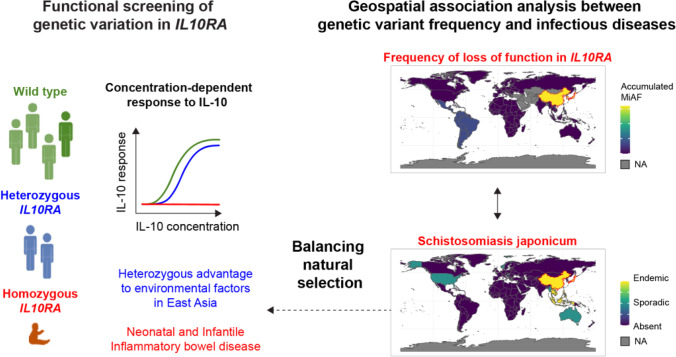

**Supplementary Information:**

The online version contains supplementary material available at 10.1007/s10875-022-01366-7.

## Introduction

Humans are exposed to numerous environmental variations such as pathogen exposure, climate, toxins, irradiation, social stress, food availability, and food quality. Natural selection of genetic variants allows adaptation to these conditions resulting in increased allele frequency of advantageous alleles and reduction in disadvantageous variants. Pathogens are considered the strongest selection pressure acting on human populations with particularly strong effects on the evolution of the immune system [1–4]. For example, *Yersinia pestis* had an impact on the TLR1-6–10 gene cluster [5] and *MEFV* that codes for the pyrin inflammasome protein [6]*.* Leprosy impacted on the evolution of the TLR1 gene [7], and tuberculosis on the WT1 gene [8]. Indeed, *Mycobacterium tuberculosis* represents a well-studied human pathogen with strong evidence for the genetic basis of susceptibility to chronic and deadly outcomes of infection [9, 10]. Due to the longer life span and chronic nature of helminth infections with co-evolutionary interaction between parasites and the human host, selective pressure of helminths on human genetic evolution has been stronger than that of viral, protozoa, or bacterial agents [1].

Natural selection of protective heterozygous variants can lead to increased disease susceptibility in individuals who are homozygous for these variants. For example, sickle cell anemia occurs in biallelic individuals from variants that confer protection against malaria in the heterozygote [11]. The equilibrium between genetic variants in the immune system is therefore not only of major importance for defining disease risk but potentially also for understanding mechanisms that could be used to develop novel treatments.

One example of the translational potential is in HIV infection. A *CCR5* loss of function (LOF) variant (CCR5-Delta32 allele) confers protection against HIV infection, and as a result, CCR5-directed stem cell transplantation or gene therapy can be used to cure HIV infection [12, 13]. This suggests that functional insight into genetic variation and respective environmental interactions is important to understand immune mediated disorders and may have clinical translational impact.

Little is known about how evolutionary mechanisms impact on susceptibility to inflammatory bowel diseases (IBD). Those disorders that are classified as Crohn’s disease (CD), ulcerative colitis (UC), and IBD unclassified (IBDu) are characterized by chronic inflammation of the gastrointestinal tract. IBD can present at any age and is increasing in prevalence and incidence worldwide [14]. Classical IBD is a multifactorial polygenic group of diseases driven by common genetic variants. These genetic risk variants for IBD are not uniformly distributed across continents. Variants in *NOD2* confer an up to 40-fold increased risk of developing Crohn’s disease in the Caucasian population [15]. The strongest IBD *NOD2* risk variant p.Leu1007ProfsTer2 is present in 2% of the European population, whereas it is present in 0.15% in South Asia and 0.05% in the East Asian population. *NOD2* variants are not only present at different allele frequencies but contribute differentially to the risk of IBD in different populations [16]. Coding variants in *IL23R* have a strong protective role against the development of IBD in individuals of Caucasian descent but not in South Asian cohorts [16]. The *ATG16L1* p.Thr300Ala variant is associated with CD in European cohorts but not within an East Asian cohort [17]. Risk variants in *TNFSF15* have a similar allele frequency in Europe and East Asia but significantly increase the risk for the development of IBD in the East Asian cohorts compared to European groups [15–17]. These examples highlight the heterogenous distribution of common genetic predisposition for developing IBD throughout the global population.

In rare cases, a single-gene defect can cause infantile IBD. Those Mendelian forms of IBD [18] often present with extreme phenotypes that are refractory to conventional medical therapy. Biallelic LOF in interleukin-10 (IL-10), IL-10 receptor subunit alpha (encoded by *IL10RA*), and IL-10 receptor subunit beta (encoded by *IL10RB*) represent genetic defects in 3 out of those monogenic disorders that cause infantile-onset IBD with high penetrance [19–21]. In patients with IL-10 signaling defects, the standard anti-inflammatory and immunosuppressive therapies used in classical IBD are often not effective as indicated by a high morbidity and mortality [19, 20, 22, 23]. Case reports and series suggest that patients with IL-10RA defects might be common in East Asia. These reports and series include the description of LOF variants in *IL10RA* p.R101W and p.T179T, that have been identified in patients in China, Japan, and South Korea [24–26]. If untreated, patients who present with IBD due to the presence of variants p.R101W and p.T179T in *IL10RA* have a very poor outcome with death in the neonatal or infantile period [24–26].

In this study, we systematically investigated the prevalence, structural, and functional consequences of pathogenic *IL10RA* variants worldwide. We analyze the global spatio-geographical distribution of infectious diseases and identify regions with high allele frequencies of variants in *IL10RA*. We pinpoint candidate infections that might drive balancing natural selection, and provide functional evidence for reduced IL-10 responsiveness in heterozygous carriers of pathogenic variants in *IL10RA*.

## Methods

### Identification of Pathogenic and Potentially Pathogenic Variants in IL-10 and Its Receptor Genes

Cases of infantile IBD due to IL-10 signaling defects were identified by literature search of infantile IBD patient cohorts. The literature is based on a recent case series [23] and complemented by an updated Pubmed search (terms “IL-10” OR “IL-10 receptor” AND “very early onset inflammatory bowel disease” OR “infantile inflammatory bowel disease”; articles published in English; last update 30th September 2019; please also see Table S3). We included pathogenic and potentially pathogenic variants published including 2020. We also included previously unpublished data from infantile onset IBD patients of the Children’s Hospital of Fudan University. We extracted the geographical origin of the patients and their variants in *IL10RA*, *IL10RB*, and *IL10*.

Non-sense LOF variants in *IL10RA*, *IL10RB*, and *IL10* (stop codon, frameshift, deletion) were identified in the Genome Aggregation Database (gnomAD). Variants classified as pathogenic were extracted from:

- Online Mendelian Inheritance in Man (OMIM, https://www.omim.org)
- Clinvar (https://www.ncbi.nlm.nih.gov/clinvar/)
- Leiden Open Variation Database (https://www.lovd.nl)

Potentially pathogenic variants were defined as (i) variants identified in patients with infantile-onset IBD and (ii) present as homozygous or combined with a compound heterozygous variant.

Pathogenic variants were defined as non-sense LOF variants (stop codon, frameshift, deletion) or previously functionally validated synonymous or non-synonymous LOF variants, (i) variants identified in patients with infantile-onset IBD, (ii) consistent with a Mendelian inheritance where biallelic variants present as homozygous or compound heterozygous, and (iii) functional evidence for LOF activity in an IL-10 signaling assay.

### Analysis of IL10RA, IL10RB, and IL10 Allele Frequencies Worldwide

We analyzed the allele frequency of pathogenic *IL10RA*, *IL10RB*, and *IL10* variants in the Genome Aggregation Database (GNOMAD), the Chinese Gene Mutation Database (disease Dx/CNGMD), the Korean Reference Genome Database (KRGDB)[27], the Human Genetic Variation Database (HGVD), and the IndiGenomes database. GNOMAD contains genome sequencing data of approximately 200,000 alleles in different populations worldwide, CNGMD v.5.0 (https://cngmd.virgilbio.com/overview) contains a sample number up to 33,372 genomes in the Chinese population, KRGDB (http://152.99.75.168:9090/KRGDB/menuPages/intro.jsp) contains whole genome sequencing data for 1722 Korean individuals, HGVD (http://www.hgvd.genome.med.kyoto-u.ac.jp) provides a resource for Japanese genetic variation (exome sequencing of 1208 individuals), and the IndiGenomes database contains whole genome sequences from 1029 individuals from diverse Indian regions (https://clingen.igib.res.in/indigen/) [28]. These data are summarized in Table S1.

### Variant Annotation and Evolutionary Analysis

Sequence analysis of the non-synonymous *IL10RA* variants was performed based on the human Interleukin-10 receptor subunit alpha *IL10RA* (Q13651-1; https://www.uniprot.org/). Structural data such as signal peptide, N-glucosylation sites, cysteine-bond, and domain structure were derived from Uniprot (https://www.uniprot.org/) or Pharos (https://pharos.nih.gov/). The JAK1 binding site [29] and E3 ligase binding sites [30, 31] were derived from the literature.

The evolutionary conservation of IL-10RA across species was analyzed using the ConSurf server [32, 33]. The IL-10RA Q13651-1 protein sequence was used as reference. Multiple sequence alignment was performed using the following settings: homolog search algorithm = HMMER; number of iterations = 1; *E*-value cutoff = 0.0001; reference database = UNIREF-90. 69 unique sequences were selected for comparison based on “Maximal %ID between sequences” = 95 and Minimal %ID for homologs = 35. The multiple sequence alignment method settings were as follows: MAFFT; calculation method = Bayesian; evolutionary substitution model = default settings (best model; JTT). The ConSurf Database tool (https://consurfdb.tau.ac.il/) was used for the structural visualization of the IL-10RA and IL-10 interaction and evolutionary conservation (PDB: 1Y6K) with default settings and based on 201 HMMER sequence hits for unique homologues.

To computationally predict variant pathogenic potential (Table S2), the REVEL [34] and CADD [35, 36] scoring systems were used.

### Structural Analysis of IL10RA Variants

The structural consequences of nonsynonymous amino acid substitutions were modeled based on the crystal structure of the human IL-10RA and interaction with IL-10 [37, 38]. Briefly, the structure (PDB: 1Y6K) was visualized within MolSoft ICM-Pro and the substitutions were independently built using the mutate residue functionality. The resulting models were then visually inspected to predict if the mutations (i) were likely to have no effect on the stability of the IL-10RA/IL-10 complex, (ii) were likely to destabilize the structure of IL-10RA, or (iii) were directly involved in IL-10RA/IL-10 interactions and therefore likely to modulate affinity between the two.

### Plasmid Isolation

pcDNA3.1( +) vector coding for wild-type or mutant IL-10RA were purchased from GenScript and expanded in Stbl3 E. coli (Thermo Fisher) according to standard protocols. Plasmids were purified using the E.Z.N.A.® Endo-Free Plasmid Maini Kit (Omega bio-tek). Following expansion in bacterial cultures and plasmid purification, all IL-10RA sequences were re-validated by Sanger sequencing (Source BioScience) to exclude mutagenesis during propagation.

### Functional Characterization of IL10RA Variants by Phosphoflow

To assess the quantitative effect of variants in *IL10RA* on IL-10 signaling, we established an in vitro assay using HEK293 cells that naturally lack *IL10RA* expression but abundantly express *IL10RB*. HEK293 cells were plated in 96-well flat bottom plates at the density of 25.000 cells/well in DMEM (Sigma) supplemented with 10% FCS (Sigma), penicillin (100 IU/mL), and streptomycin (100 μg/mL) (Sigma-Aldrich). Following 24 h, cells were transfected with the ratio 0.2 µg DNA/0.4 µL Lipofectamine2000 per well in Optimem media (Gibco). A GFP-encoding plasmid was co-transfected to allow gating on successfully transfected cells. All transfections showed comparable efficiency based on the GFP signal. 24 h post transfection HEK293 cells were serum starved in plain DMEM for 2 h. Following starvation cells were stimulated in DMEM with recombinant human IL-10 (Peprotech) or universal type I interferon (pbl assay science) for 15 min. After stimulation cells were washed in PBS (Sigma-Aldrich) and stained on ice using the Fixable Viability Dye eFluor® 780 (eBioscience) for 15 min to exclude dead cells from the analysis. Cells were then washed with PBS supplemented with 0.5% FCS (Sigma-Aldrich) and fixed by incubation in 3.7% Formaldehyde (Sigma-Aldrich) at 37 °C for 20 min. Permeabilization was performed on ice for 30 min in − 20 °C 90% methanol (Merck). Staining was performed for 1 h at room temperature in PBS supplemented with 0.5% FCS using the Alexa Fluor 647-conjugated antibody anti-phospho-STAT3 (pY705) (Becton Dickinson (BD); clone 4/P-STAT3). Samples were acquired on a LSRII (BD) flow cytometer and analyzed using FlowJo version 10.6.1 (BD).

### PBMC Isolation, Cell Culture, and STAT3 Phosphoflow

EDTA-anticoagulated blood was diluted with an equal volume of phosphate-buffered saline (PBS). PBMC were obtained by density gradient centrifugation (TBDsciences, LTS1077, China). PBMC were washed twice in PBS, and plated in 96-well flat bottom plates at the density of 5 × 10^5^ cells/well in RPMI 1640 (Sigma) supplemented with 10% fetal bovine serum (Gibco), 1% penicillin, and streptomycin. Stimulations with recombinant human cytokines (IL-10 (R&D Systems; Cat 217-IL-005), IL-6 (Peprotech; Cat 200–06), Universal type I interferon (pbl assay science; Cat 11,200–1)) were performed for 15 min after resting PBMC for 40 min. Following, cells were washed in Stain Buffer (BD Pharmingen; Cat 554,656) and stained using the APC-H7 Mouse Anti-Human CD3 (BD Pharmingen; Cat 560,176; Clone: SK7), FITC Mouse Anti-Human CD4 (BD Pharmingen; Cat 555,346; Clone: RPA-T4), and PE Mouse Anti-Human CD14 (BD Pharmingen; Cat 555,398; Clone: M5E2) for 15 min at 4 °C. Cells were then washed with Stain Buffer and fixed using Fix Buffer I (BD Phosflow; Cat 557,870) at 37 °C for 10 min. Permeabilization was performed on ice for 30 min in Perm Buffer III (BD Phosflow; Cat 558,050). Staining was performed for 1 h at room temperature in Stain Buffer using the Alexa Fluor 647-conjugated antibody anti-phospho-STAT3 (pY705) (BD Pharmingen; Cat 557,815; Clone: 4/P-STAT3) and Alexa Fluor 647 Mouse IgG2a, κ Isotype Control (BD Phosflow; Cat 558,053; Clone: MOPC-173). Samples were acquired on a FACS Canto II flow cytometer (BD) and analyzed with FlowJo version 10.6.1 (BD).

### Spatial Analysis of Infection Disorders

We searched the Global Infectious Diseases and Epidemiology Network (GIDEON) database for all available disorders in GIDEON online (retrieved from www.gideononline.com on 23.08.2018. A pathogen search across 229 countries or regions in the GIDEON database identified 357 infectious diseases. 188 diseases were excluded because of unspecific terms, worldwide endemic distribution, or not endemic to any region. In total, 169 infectious diseases were analyzed. Prevalence of pathogens within each country or region was coded as not present in humans (code 0.001), rare occurrence (code 100), and endemic or potentially endemic (code 200). For the spatial correlation analysis between disorders with variant enrichment, global occurrences were excluded. For downstream analysis, the data was processed and visualized using R (version 3.6.3) and RStudio (version 1.2.5001). Per-country disease presence codes were normalized and scaled with the Seurat R package (version 3.1) [39, 40]. Variable diseases were identified using the “FindVariableFeatures” function. All 169 diseases were used as input for principal component analysis (PCA). 24 PCs were selected for principal component analysis following ranking of principle components based on the percentage of variance explained using the “ElbowPlot” function. A shared nearest neighbor graph was constructed using the “FindNeighbors” function over the first 24 dimensions. Optimal resolution for Graph-based clustering was determined using the Clustree function [41] allowing the visualization of a range of resolutions (0.2–4) and performed on 24 components at a resolution of 2.6. Cluster-associated markers were identified using the Seurat “Findmarkers” function (mean log2 fold change > 2). The uniform manifold approximation and projection (UMAP) (Fig. 6A and Supplementary Fig. 6B) was computed using the first 24 reduced dimensions. Identified clusters of countries were mapped onto the world map using the R packages Googleway (version 2.7.3), ggspatial (version 1.15), lwgeom (version 0.2–5), Simple Features for R (version 0.9–8), rnaturalearth (version 0.1.0), and rnaturalearthdata (version 0.1.0).

### Classification of Candidate Infectious Disorders

The functional association between each infectious disorder and IL-10 signaling was classified as strong when evidence in the literature suggested an association between infection and increased IL-10 in humans and functional mouse models suggest that blockade of IL-10 signaling by gene targeting or monoclonal antibodies prevented immunopathology and improved outcome. Evidence was rated as moderate when either in vitro or and human data suggest an association, but no functional data were available.

### Statistics

Statistical analyses were performed with GraphPad Prism, version 8.0 for Macintosh (GraphPad Software, La Jolla, CA) or Microsoft Excel for Mac, version 15.32. *P*-values ≤ 0.05 were considered significant and indicated as follows: *P ≤ 0.05; **P ≤ 0.01; ***P ≤ 0.001; ****P ≤ 0.0001. Statistical tests are described in figure legends.

## Results

### An Atlas of the Genetic Architecture of IBD and Human Pathogenic Variants in the IL-10 Signaling Pathway

**Fig. 1:**
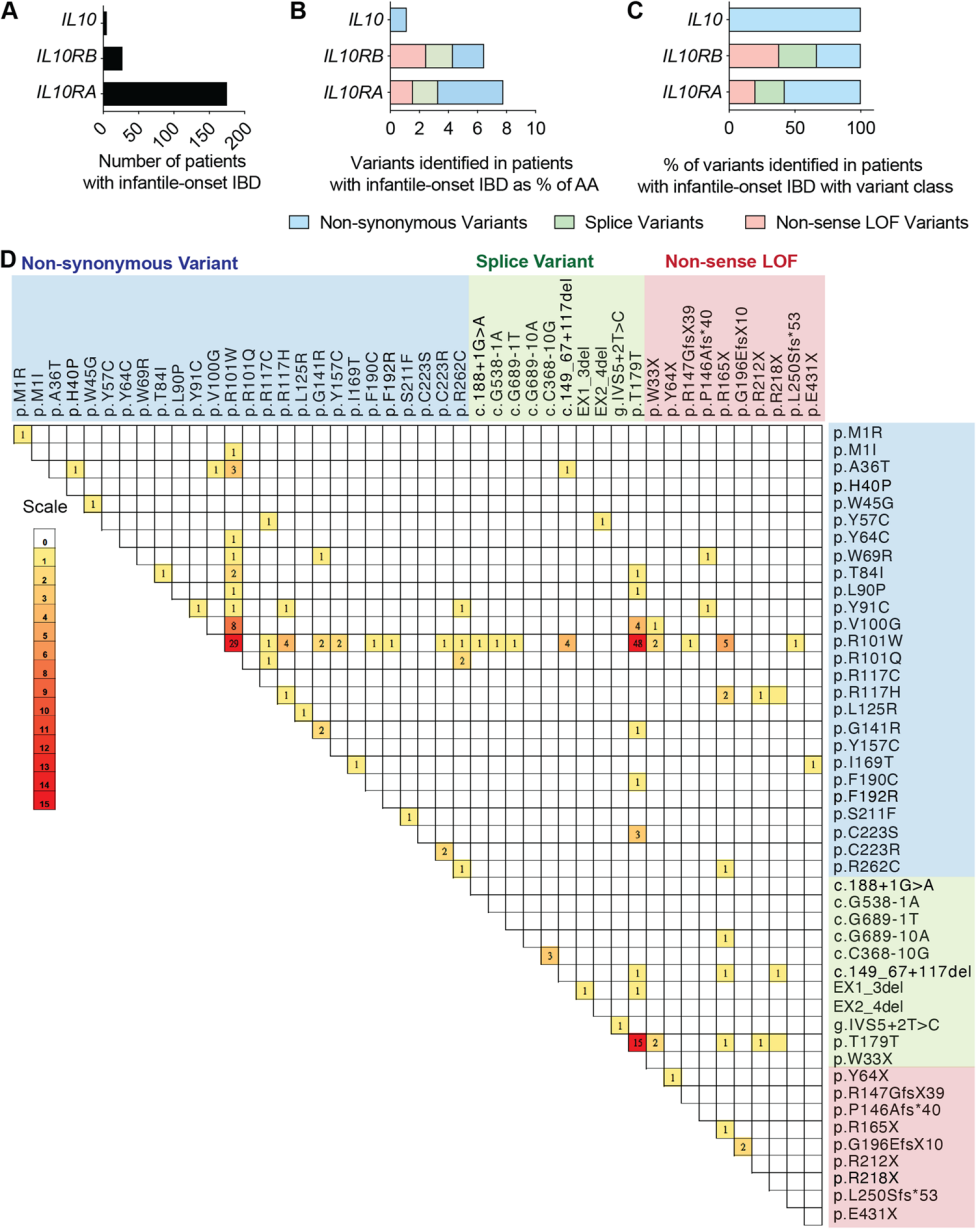
Global classification of *IL10RA* variants. **A** Number of patients with *IL10*, *IL10RA*, and *IL10RB* defects presenting with infantile-onset IBD. **B** Number of LOF variants present in the *IL10*, *IL10RB*, and *IL10RA* gene expressed as percent of amino acid length. **C** Percent of patients with LOF variant class. **D** Distribution of variants among patients with infantile-onset IBD. Scale shows the number of patients with the variant

We performed a comprehensive search of all pathogenic or potentially pathogenic variants in *IL10RA*, *IL10RB*, and *IL10* in published literature, large cohorts of infantile IBD, clinical genetics databases, and complemented this with a search of non-sense LOF variants in population-based databases (gnomAD). Among the 207 reported patients with infantile IBD caused by IL-10 signaling pathway defects, the majority present with *IL10RA* deficiency (*n* = 175; 84.5%), followed by *IL10RB* (*n* = 27; 13.0%), and *IL10* defects (*n* = 5; 2.5%) [23] (Fig. 1A; Table S1; Table S2). Overall, we identified 67 confirmed or potentially pathogenic variants (*IL10RA*, *n* = 45; *IL10RB*, *n* = 20; *IL10*, *n* = 2). While the two variants in *IL10* that have been identified in patients with infantile-onset IBD are both non-synonymous variants, the sequence length-normalized proportions of non-sense LOF variants and confirmed splice variants in *IL10RB* and *IL10RA* are 2.5% and 1.8%, and 1.6% and 1.7%, respectively (Fig. 1B). In comparison, there are low sequence length-normalized and non-normalized proportions of non-synonymous variants in *IL10RB* (2.2%, 33.3%) compared to *IL10RA* (4.5%, 57.8%) (Fig. 1B and C). Unlike many other recessive disorders where there is a high proportion of consanguineous families and a substantial proportion of patients present with homozygous variants, a large proportion of patients with potentially pathogenic variants in *IL10RA* are compound heterozygous (Fig. 1D). 60% of the variants in *IL10RA* and 92% of patients have not been functionally characterized (Supplementary Fig. 1).

Because of the non-synonymous nature of the majority of the variants and the combinatorial variation among patients, this means that the functional impact of the genetic variants is difficult to predict precisely and need to be validated either individually using primary patient derived cells or in a standardized assay.

### Functional Characterization of IL10RA Non-synonymous Variants

In light of the large number of functionally uncharacterized variants that were found in patients with infantile IBD, we performed a systematic functional characterization of those potentially pathogenic *IL10RA* variants in HEK293 cells. HEK293 cells express endogenously *IL10RB* but not *IL10RA* (Supplementary Fig. 2A). In agreement with the gene expression of IL-10 receptor chains, wild-type HEK293 cells only responded to IL-10 stimulation once transfected with wild-type *IL10RA* (Supplementary Fig. 2B). Irrespective of the experimental conditions (non-transfected, transfected with an empty vector, or transfected with a vector coding for wild-type *IL10RA*), HEK293 cells responded comparably to type I interferon (IFN) demonstrating intact and comparable STAT3 phosphorylation (Supplementary Fig. 2B).

**Fig. 2:**
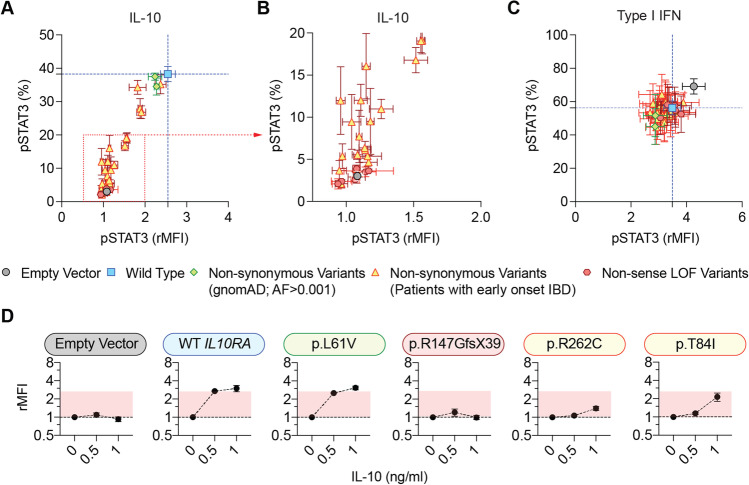
Functional characterization of *IL10RA* variants. Summary of IL-10-induced (0.5 ng/mL) (**A** and **B**) and (**C**) Type I interferon-induced (10^4^ units/mL) (right panel) STAT3 phosphorylation in HEK293 cells transfected with and empty vector, wild-type *IL10RA* or distinct variants in the *IL10RA* gene covering non-sense LOF variants, infantile-onset IBD-associated non-synonymous variants, and two variants not associated with disease (Table S2) as measured by flow cytometry. Relative mean fluorescence intensity (rMFI) to untreated cells is shown on the *x*-axis and % pSTAT3 positive gated cells on the *y*-axis. The mean ± SD of two independent experiments and each 2 to 4 technical replicates is shown. **D** Individual examples of IL-10-induced STAT3 phosphorylation (rMFI) at the baseline or following stimulation with 0.5 and 1 ng/mL exogenous cytokine. Each data point indicates the mean ± SD of 2 to 3 independent experiments and each 2 to 4 technical replicates

We performed a qualitative and quantitative analysis of IL-10-induced signal transducer and activator of transcription (STAT) 3 phosphorylation among 24 non-synonymous *IL10RA* variants, 6 non-sense LOF *IL10RA* variants, and wild-type *IL10RA.* As a benchmark, we included 8 previously validated LOF variants in *IL10RA* (Table S3). Furthermore, we included 2 non-synonymous potentially non-pathogenic variants in *IL10RA* that have a higher allele frequency than 0.001 in the gnomAD database, rendering a LOF effect unlikely (p.L61V, p.V113I). Additionally, we analyzed a recently described rare variant in *IL10RA* that has been identified by association studies, p.P295L [42]. Empty vector transfected and IL-10 stimulated HEK293 cells served as negative control, and STAT3 phosphorylation induced by type I interferon stimulation served as positive control. Interestingly, HEK293 cells transfected with the polymorphisms p.L61V and p.V113I, or p.P295L showed IL-10-induced pSTAT3 responses similar to wild-type *IL10RA* (Fig. 2A; Fig. 2D; Supplementary Fig. 3A; Supplementary Fig. 4). At 0.5 ng/mL IL-10 stimulation, 24 variants that were identified in patients with infantile-onset IBD, including the 8 previously validated LOF variants in *IL10RA*, showed a statistically significant decrease STAT3 phosphorylation, demonstrating LOF. 6 variants, 1 located in the signal peptide (p.M1I), and 5 non-synonymous variants (p.A36T, p.Y57C, p.W69R, p.R101Q, p.I169T) showed a trend towards reduced responses to IL-10 stimulation, but these differences did not reach statistical significance of *p* < 0.05 in the HEK293 cell system (Fig. 2A; Supplementary Fig. 3A). Across tested concentrations, 13 out of 34 tested variants showed statistically significant reductions in IL-10 signaling at 1 ng/mL IL-10 stimulation, and 7 out of 34 tested variants showed a statistically significant reduction in IL-10 signaling at 10 ng/mL IL-10 stimulation (Supplementary Fig. 3A). Importantly, type I IFN responses were not affected (Fig. 2C; Supplementary Fig. 3B). Together these results functionally validate 8 known and 22 uncharacterized variants in *IL10RA*, provide a hierarchy of the impact on IL-10 signaling, and identify and validate 16 previously uncharacterized LOF variants in IL-10RA.

### Pathogenic Variants in the IL10RA Gene Affect Critical Cytokine Binding Domains

**Fig. 3:**
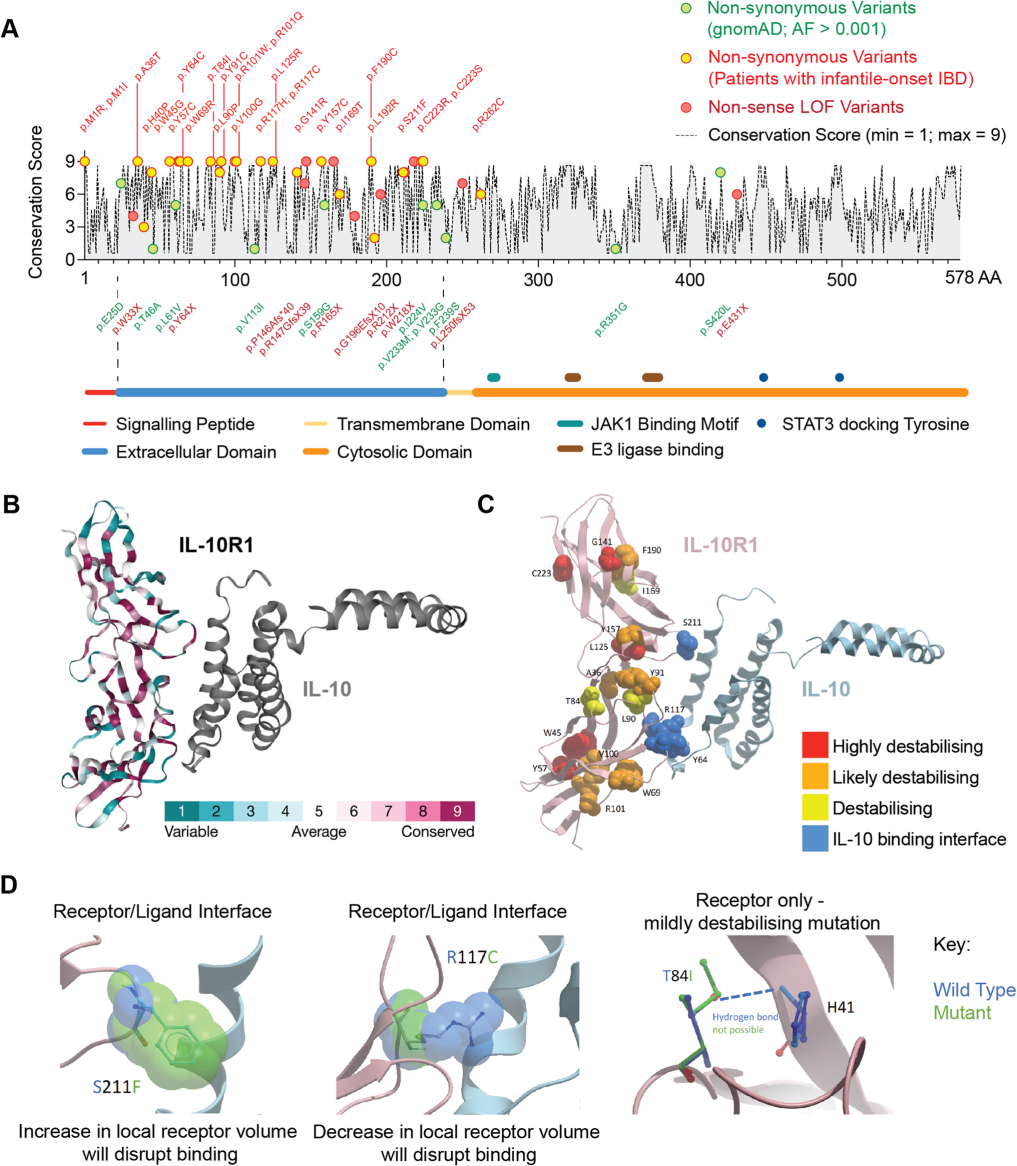
Conservation and structure of the IL-10RA. **A** ConSurf server-based analysis of the evolutionary conservation of IL-10RA (Q13651-1) across 69 HMMER-identified homologues. Non-synonymous variants and non-sense LOF variants position in the amino acid sequence are indicated. Domain structures with signal peptide, extracellular domain, transmembrane domain, cytosolic domain, JAK1 binding motif, E3 ligase binding sites, and STAT3 docking tyrosines are shown. **B** Structural model (PDB: 1Y6K) of the human IL-10RA extracellular domain interacting with human IL-10. The ConSurf conservation across the amino acid sequence is indicated based on 201 HMMER-identified homologues. **C** Structural presentation showing the location of pathogenic variants and their predicted impact on protein structure and IL-10RA–IL-10 interaction. **D** Close-up view on the predicted structural impact of 2 pathogenic variants in IL-10RA affecting cytokine receptor–cytokine interaction (p.S211F and p.R117C), and one example of a pathogenic variant that internally affects the IL-10RA structure (p.T84I)

We next investigated the potential structural consequences of pathogenic variants in *IL10RA*. We focused on non-synonymous variants assuming that stop-loss or frameshift LOF variants as well as pathogenic splice variants will have similar detrimental effects irrespective of the position within the gene. The non-synonymous variants in *IL10RA* are enriched in the extracellular domain irrespective whether normalized to the length of the molecule or not. 26 of the 27 non-synonymous variants are located in the extracellular domain that accounts for 214 amino acids (22–235) of 578 amino acids (Fig. 3A). The majority of these variants involve highly conserved regions in critical functional sites of IL-10RA (Fig. 3A–C). Three of the five interaction sites [37] critical for binding of the cytokine IL-10 to IL-10RA are affected by pathogenic variants in *IL10RA*. This includes the contact amino acid residues of IL-10RA p.Y64C, p.R117C, and p.S211F (highlighted in Fig. 3C and D). Multiple pathogenic *IL10RA* variants disrupt secondary structures of the extracellular domain such as beta strands (p.A36T, p.W45G, p.Y57C, p.I169T, p.V100G, p.R101W, p.R101Q, p.L125R, p.G141R, p.F190C, p.C223S, p.C223R) or alpha-helixes (p.L90P, p.Y91C, p.T84I, p.Y157C) (Fig. 3C). The variants p.C223S and p.C223R will disrupt the critical disulphide bond between C202 and C223. Additional variants disrupt the signal peptide (p.M1I and p.M1R). These findings suggest that the vast majority of pathogenic non-synonymous genetic defects affect structural elements that are critical for the IL-10RA extracellular structure and cytokine binding.

### Geographical Cluster of Patients with IL10RA Defects in East Asia

**Fig. 4:**
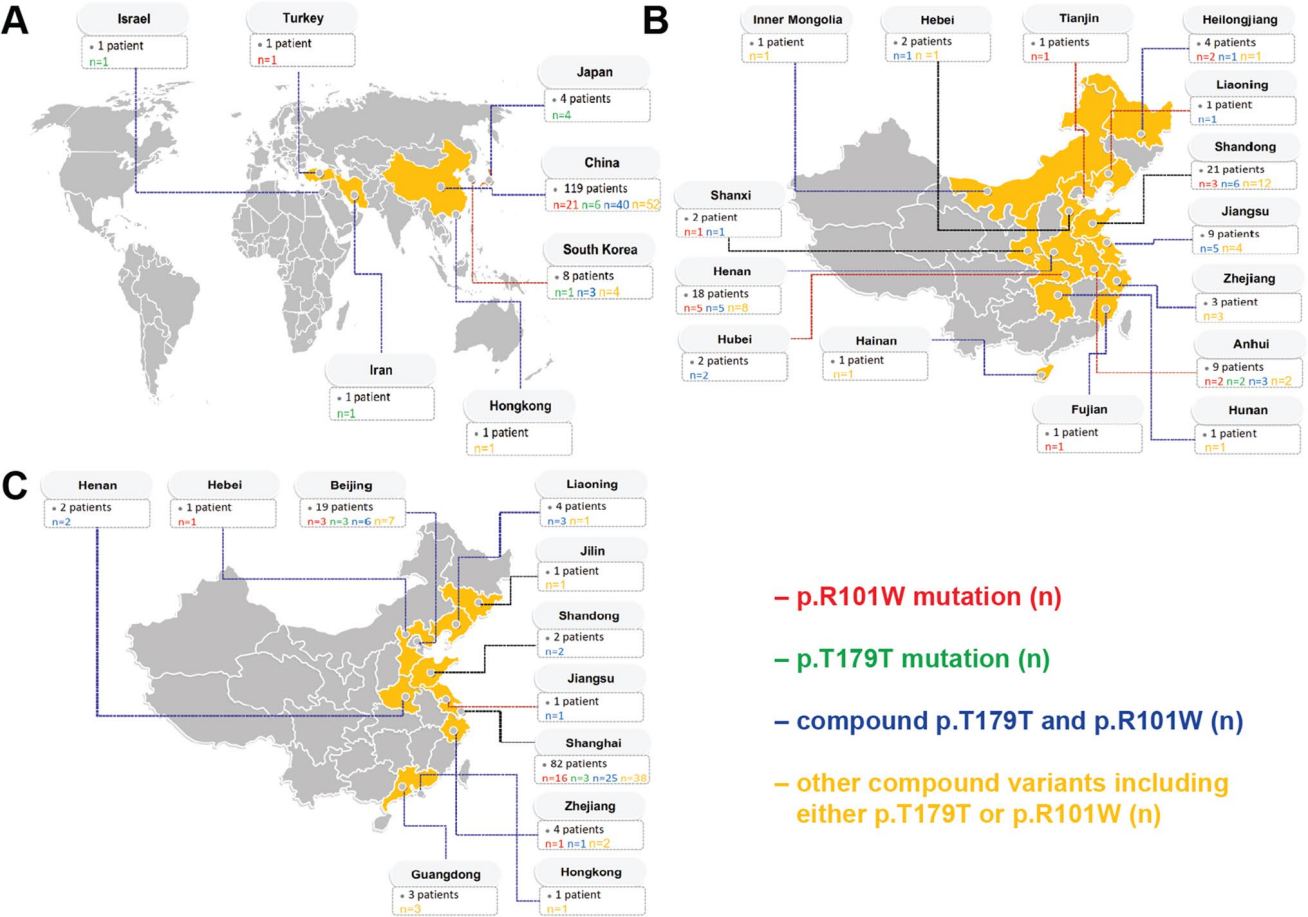
Geographical distribution of patients with pathogenic *IL10RA* variants. **A** Worldwide distribution of infantile IBD patients with *IL10RA* variants (origin). **B** Infantile IBD patients with IL-10RA defects reported by pediatric gastroenterology centers in China. **C** Origin of infantile IBD patients with *IL10RA* variants that were treated at the Children’s Hospital of Fudan University

**Fig. 5:**
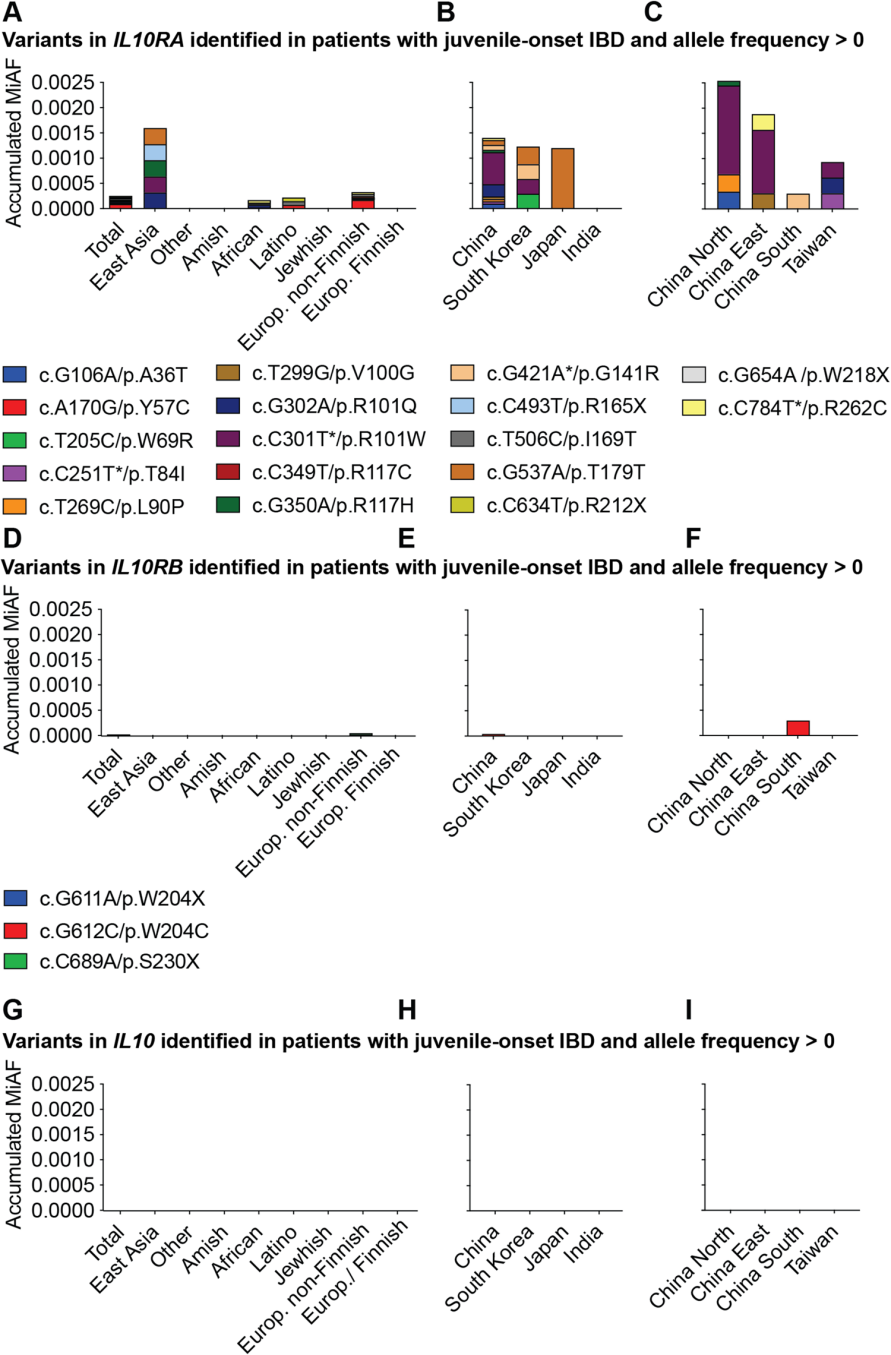
Geographical distribution of pathogenic variants in *IL10RA*, *IL10RB*, and *IL10*. **A** Allele frequencies of pathogenic variants in *IL10RA* in different populations/continents of the world (carrier). **B** Allele frequencies of pathogenic variants in *IL10RA* China, South Korea, Japan, and India. **C** Allele frequencies of pathogenic variants in *IL10RA* within different regions in China and Taiwan. Staged bars show aggregated allele frequencies and the respective variants. **D**–**F** Allele frequencies of pathogenic variants in *IL10RB* and **G**–**I** *IL10* in different populations/countries of the world including China, South Korea, Japan, India, and different regions in China and Taiwan. All variants in databases were found as heterozygous carrier status. Only variants with allele frequencies > 0 are shown. For the complete list of allele frequencies, please refer to Table S4

There are substantial differences in the geographical distribution of non-sense LOF defects in *IL10RA*, *IL10RB*, and *IL10* among different patient cohorts worldwide (Figs. 4A, 5A, 5D, and 5G; Table S1). The highest numbers of patients with infantile IBD due to *IL10RA* defects are found in East Asia (or East Asian descent) (Fig. 4A; Table S1). In contrast, the majority of patients with *IL10RB* defects descent from Central and South Asia as well as Europe (Turkey, Bangladesh, Pakistan, Sicily, Germany, Poland, France; Table S1) and derive from consanguineous families (Zheng et al. 2019 [23] and unpublished results).

Importantly, geospatial analyses of age and gender-adjusted prevalence rates of IBD by the Global Burden of Disease 2017 Inflammatory Bowel Disease Collaborators [43] have not revealed an enrichment in East Asia, suggesting that IBD per se is not of high prevalence in the East Asian region although it has been increasing over the previous decades, but the monogenic condition *IL10RA*-associated infantile enterocolitis has highest absolute case numbers reported worldwide in the East Asian region (China, Japan and South Korea). This suggests that biallelic variants cause a high prevalence of monogenic infantile IBD but that the heterozygote MAF of those variants unlikely has a strong population effect. This is in keeping with the observation that the heterozygous patient parents of children with IL10RA defects do not develop intestinal inflammation.

In East Asian patients with infantile IBD, multiple pathogenic *IL10RA* variants were detected (Fig. 4B and C). 99.1% of patients with the *IL10RA* p.R101W variant and 97.0% of patients with *IL10RA* p.T179T variant are located in East Asia (Fig. 4A). Within China, and in particularly in the eastern part of China, infantile IBD patients with pathogenic *IL10RA* variants were reported by multiple centers. To exclude those patients genetically originated from a single area, we identified the patients’ place of birth. These analyses demonstrated that patients with pathogenic *IL10RA* variants originated from different Chinese regions (Fig. 4C).

### Geographical Accumulation of Pathogenic IL10RA Variants in East Asia

The geographic cluster of patients with *IL10RA* defects and infantile IBD in East Asia raises the question whether this finding is due to the population size in China, recent awareness of the condition, due to local founder effects, or due to an accumulation of pathogenic variants as part of an evolutionary enrichment. We therefore analyzed the population allele frequencies of pathogenic *IL10RA* variants identified in different geographic regions (Fig. 5A–C) and compared these to the distribution of pathogenic *IL10RB* and *IL10* variants (Fig. 5D–I). 17 pathogenic *IL10RA* variants can be found in population databases (others are too rare to be sampled based on currently available sequencing data). The highest accumulation of pathogenic *IL10RA* variants is present in North and East China. The 7 most common variants in those regions each have a higher allele frequency compared to the aggregated allele frequencies of pathogenic *IL10RA* variants in all other populations worldwide (Fig. 5A).

We next investigated population allele frequencies within East Asia. Although China, South Korea, and Japan have a similar overall aggregated allele frequency, the variant distribution is significantly different (Fig. 5B and C). p.R101W, the most common variant in China (MAF 0.0635%), is absent in Japan. In contrast, the most common variant in Japan p.T179T is significantly less frequent in China (MAF 0.12% versus 0.0096%, respectively). Furthermore, significant differences are present in respect to the geographic distribution of pathogenic *IL10RA* variants within China (Fig. 5C). The highest aggregated MAF of pathogenic variants is found in North and East China with 7 variants contributing to this enrichment (Fig. 5C; Table S1).

The enrichment of 12 independent pathogenic *IL10RA* variants across East Asia and their broad geographical distribution argue against a limited regional founder effect and suggest that evolutionary selection pressure maintains these variants within the population. In summary, regional differences suggest that variants have expanded in different regions.

### Geospatial Association Analysis Between Pathogenic IL10RA Variants and Human Pathogen Distribution Worldwide

**Fig. 6:**
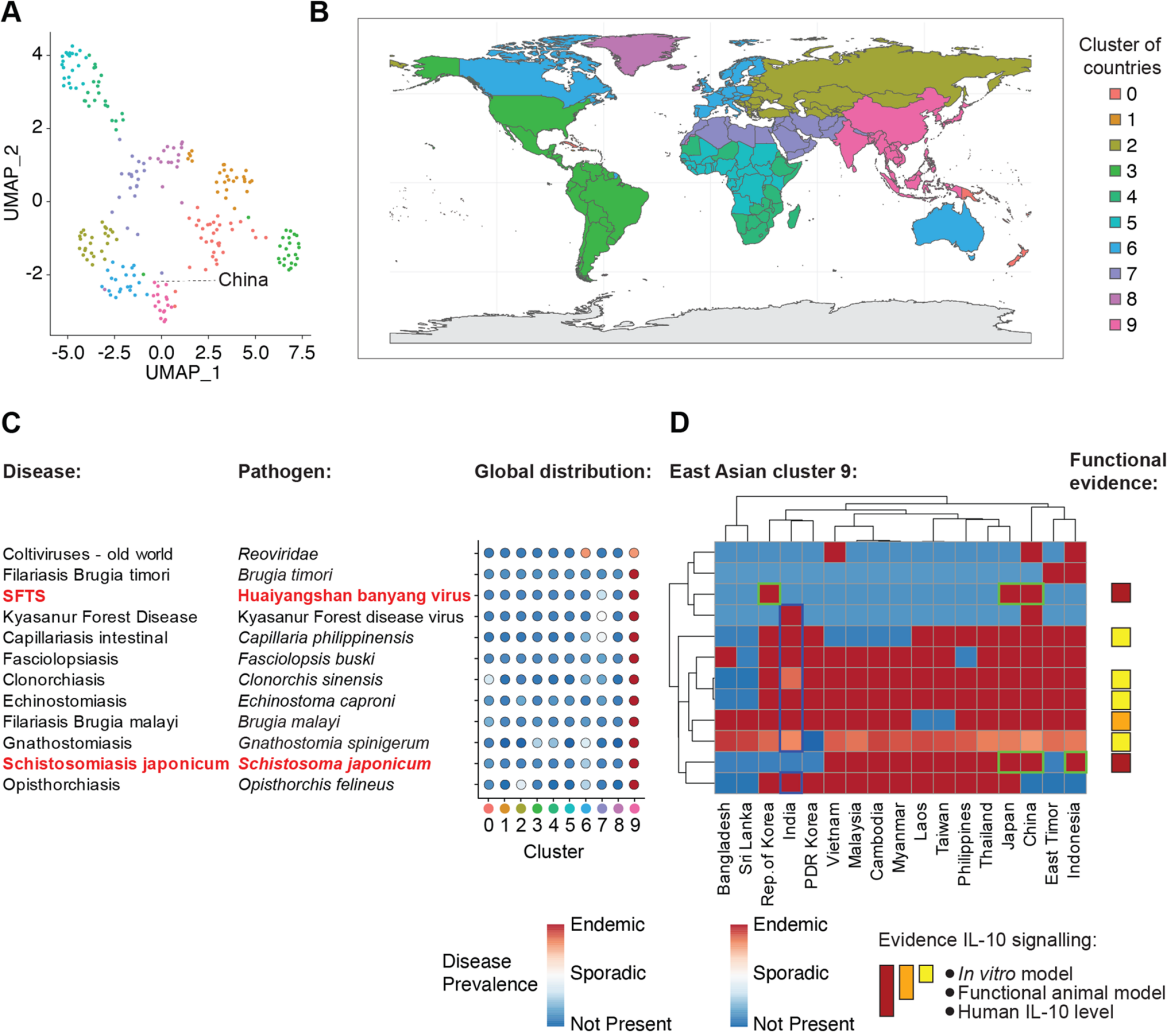
Geospatial association of infectious disorders in East Asia with pathogenic *IL10RA* variants. **A** UMAP presentation of identified clusters of countries based on the distribution (not present, sporadic, endemic) of 169 diseases. **B** Worldmap showing the geographical distribution of clusters of countries according to **A**. **C** Dotplot presentation of those diseases that were found to be associated (mean log fold change > 2) with cluster 9 representing the region of interest. **D** Heatmap presentation of cluster 9 countries and cluster-specific diseases, and literature-based evidence for a beneficial role of IL-10 signaling inhibition in respective pathogen clearance (functional evidence color code: red indicates 3 levels of evidence; orange indicates 2 levels of evidence, and yellow indicates one line of evidence)

To identify pathogens that could be acting as selection pressure on the *IL10RA* gene, we performed an unbiased geospatial association study. We selected all 357 pathogens in the GIDEON (www.gideononline.com) database (Supplementary Fig. 5). We hypothesized that evolutionary selection is likely driven by pathogens endemic to the geographic distribution of the East Asian region corresponding to the increased allele frequency of pathogenic *IL10RA* variants. In the absence of allele frequency data in several countries such as Mongolia or the Siberian region of Russia, we assumed that the core region would involve North and East China, Korea, and Japan but would not necessarily be restricted to this region. To identify those diseases that are the source of variation in the data set and specifically enriched in the region of interest, we performed linear dimensionality reduction and uniform manifold approximation and projection (UMAP) of infections in individual countries worldwide [39, 40] (Fig. 6A). We identified 10 clusters of countries based on the absence, sporadic presence, or endemic presence of a given disease (Fig. 6A and B). China was located in cluster 9 consisting of 17 countries (Fig. 6A–D). We identified 12 infectious disorders specifically associated with cluster 9 (Gnathostomiasis, Fasciolopsiasis, Filariasis Brugia malayi, Echinostomiasis, Clonorchiasis, Filariasis Brugia timori, Kyasanur forest disease, Opisthorchiasis, Coltiviruses — old world, Schistosomiasis japonicum, Capillariasis intestinal, and severe fever with thrombocytopenia syndrome (SFTS)) (Fig. 6C and D; Supplementary Fig. 6A and 6B). Among those diseases associated with cluster 9, 2 were not present in China, 1 was listed as sporadic, and 9 were endemic (Fig. 6D). Interestingly, 7 out of these 9 diseases are also endemic to India, where no increased frequencies for pathogenic variation in *IL10RA* have been documented (Figs. 5D–F and 6D). The remaining two candidate disorders are SFTS caused by phlebovirus infection of the *Bunyaviridae* family and Schistosomiasis japonicum caused by infection with the *Schistosoma japonicum* parasite.

Next, we identified plausible candidate disorders that might drive natural selection among those cluster 9-associated diseases. Strong evidence that IL-10 signaling restricts host protective immune responses is available for phlebovirus infection of the *Bunyaviridae* family and Schistosomiasis japonicum (*Schistosoma japonicum*). In vitro experimental evidence additionally suggests a beneficial role for reduced IL-10 signaling in diseases including Filariasis (*Brugia malayi*), Capillariasis intestinal (*Capillaria philippinensis*), Opisthorchiasis (*Opisthorchis felineaus*) Clonorchiasis (*Clonorchis sinensis*), and Echinostomiasis (*Echinostoma caproni*) [44–55] (Fig. 6C). Importantly, the recent emergence of SFTS in rural areas of China [56, 57] suggest that this infection is not a plausible candidate, whereas Schistosomiasis japonicum, that has been endemic to China for several thousands of years [58], is the most likely source of long term pathogen-mediated selection pressure.

### Pathogenic Variants in IL10RA Reduce IL-10 Responsiveness in the Heterozygous State

**Fig. 7:**
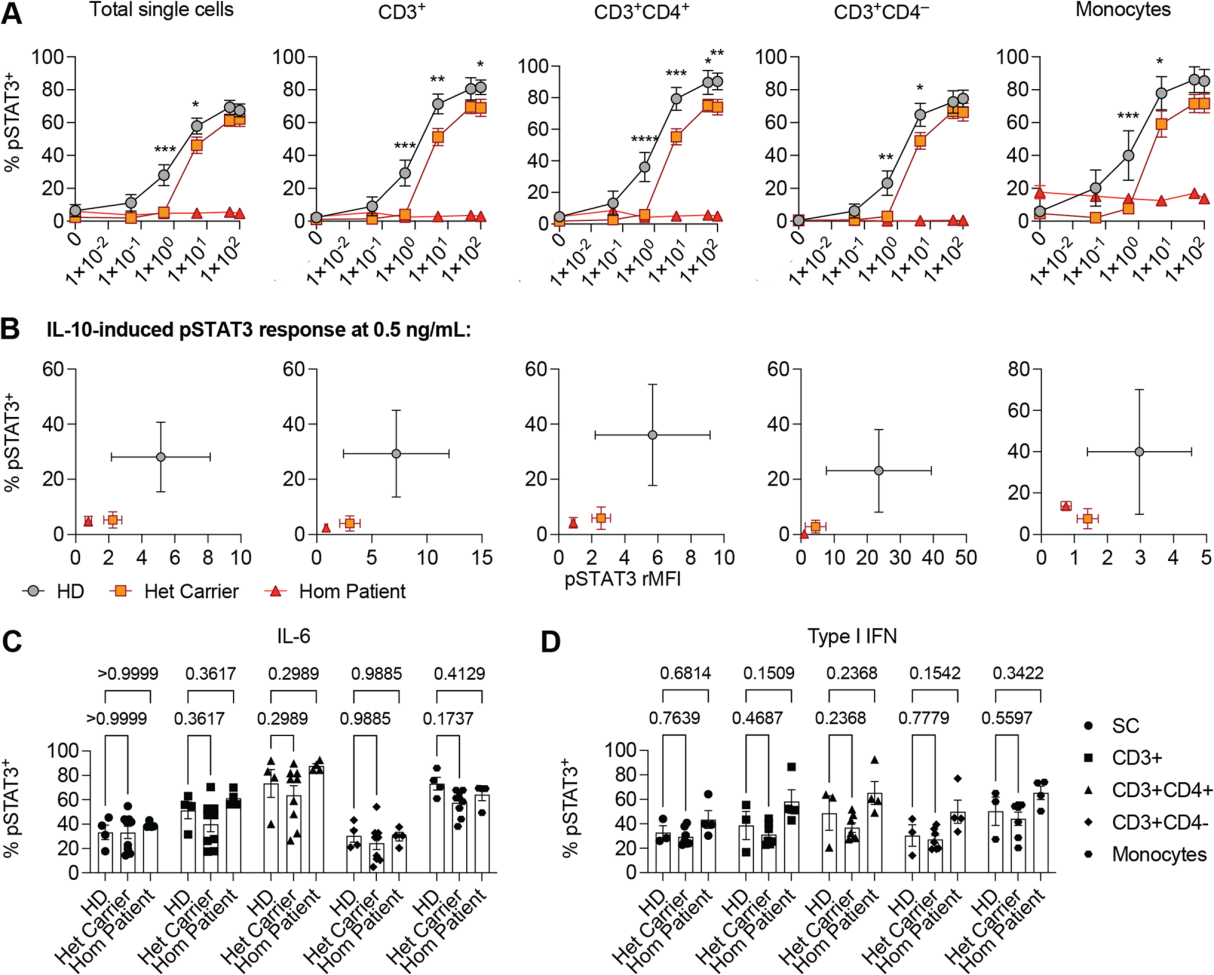
IL-10 responses are reduced in heterozygous carriers of pathogenic *IL10RA* variants. **A** Line plots show IL-10-induced STAT3 phosphorylation in PBMC from healthy non-carriers (*n* = 4), healthy heterozygous carriers (*n* = 9, p.V100G, p.W33X, p.R101W, p.T179T), and 3 patients with compound heterozygous carrier state of a pathogenic variant in *IL10RA* (P1: p.V100G/p.T179T; P2: p.W33X/p.T179T; P3: p.R101W/p.T179T) (Mann–Whitney test). **B** *XY*-graphs show the quantitative (*x*-axis, rMFI) and qualitative (*y*-axis, % pSTAT3^+^) response to stimulation with 0.5 ng/mL IL-10 according to **A**. **C** The bar graphs show the pSTAT3 response to IL-6 (100 ng/mL) and Type I IFN (10.^4^ U/mL) stimulation across diverse lymphocyte populations (Mann–Whitney test)

The enrichment of pathogenic *IL10RA* variants across East Asia and characteristic endemic presence of infectious disease with known functional link to the IL-10 signaling pathway led us to hypothesize that these variants mediate environment-specific increased fitness in heterozygous carriers. If this hypothesis is correct, one would hypothesize that heterozygous variants would have a mild but measurable functional impact on IL-10 signaling. We stimulated PBMC from healthy controls, healthy heterozygous *IL10RA* variant carriers (parents) and homozygous infantile patients with IBD with IL-10 or type I interferon and measured STAT3 phosphorylation. The heterozygous carriers showed a shifted dose response curve and significantly decreased responses towards IL-10 stimulation in total PBMC, gated T cells, and gated monocytes. These responses were absent in PBMC from homozygous patients (Fig. 7A and B). Importantly, IL-6 or type I interferon-induced phosphorylation of STAT3 was similarly detected in all 3 groups (Fig. 7C and D). These results demonstrate a functional impact of the heterozygous carrier state of pathogenic variants in *IL10RA* on IL-10 signaling and support the hypothesis that modest heterozygous effects might have driven balancing natural selection due to heterozygous advantage in the context of environmental evolutionary pressure.

## Discussion

We have summarized the spectrum of IL10RA variants associated with infantile onset IBD and systematically validated multiple pathogenic IL10RA variants. Our analysis of IL-10 responses across a range of concentrations in primary immune cells and HEK293 cells underlines the dynamic contribution of the IL10A variants. Failure to detect a functional effect of the GWAS-identified variant p.P295L may indicate a more subtle modulatory effect that may be relevant in the context of polygenic risk. We have focused our functional analysis on variation of the canonical variant of the *IL10RA* gene (ENST00000227752). Whether the shown functional effects solely affect IL-10 binding, as suggested by the modeling studies, or are at least partially caused by changes in protein expression cannot be excluded.

Biallelic LOF variants in *IL10RA* cause significant health problems for the small number of children worldwide. These children require genomic screening and personalized medicine with allogenic hematopoietic stem cell transplantation [18, 20, 23]. Therefore, our population-based studies on pathogenic *IL10RA* variants have implications for health care provision. A simplified model of equal distribution of the pathogenic *IL10RA* variants throughout China, with a combined MAF of approximately 0.14% would mean that 1 in 2.5 million children carry biallelic variants and present with neonatal and infantile IBD. Due to the high frequency of pathogenic *IL10RA* variants in North China (0.3%), this accounts for approximately 1 in 400,000 new-born in this region and likely accounts for the highest incidence of neonatal IBD worldwide.

Although studies on the evolution of cytokines and their receptors suggest that *IL10* and *IL10RA* may be subject to natural selection in response to pathogens, especially parasites [59–61], balancing selection of pathogenic genetic variants has not specifically been demonstrated. Our data suggest that accumulation of pathogenic *IL10RA* variants is not due to a limited “founder effect” and geographical isolation. Balancing natural selection is supported by several lines of evidence: (i) despite complete penetrance of a lethal phenotype in the biallelic setting; (ii) there is geographically restricted accumulation of multiple pathogenic *IL10RA* variants that cause defective IL-10 signaling, some protein coding defects encoded by two different DNA variants; (iii) with a wide distribution in the East Asian region; and (iv) biologic plausibility of potentially beneficial heterozygote effects according to our current understanding of *Schistosoma japonicum* infection pathogenesis in human and animal models.

Interestingly, in *IL10RB*, a comparable accumulation of pathogenic variants was not identified. This likely reflects the role of IL-10RB in mediating signaling across a range of cytokines (IL-10, IL-22, IL-26, IL-28A, IL-28B, and IL-29).

Schistosomiasis japonicum, one of the most debilitating and mortal parasitic diseases in the world, likely causes a strong selection pressure during human evolution in East Asia. An example of likely protective host adaptation to *Schistosoma japonicum* is cholesteryl ester transfer protein (encoded by *CETP*) deficiency, which is common in endemic regions of East Asia. *Schistosoma japonicum* requires normal plasma HDL for the embryonation of their eggs [62], the pathway that is defective in cholesteryl ester transfer protein deficiency. Adaptation to IL-10 receptor defects might confer a similar host protective adaptation to *Schistosoma japonicum*. Animal models support a direct pathogenic role of IL-10. CD4^+^ T cells express high IL-10 levels in the C57BL/6 mouse liver during *Schistosoma japonicum* [46]. A recombinant protein (cercarial secreted Sj16 protein) derived from *Schistosoma japonicum* stimulated IL-10 production, inhibited LPS-induced bone marrow-derived dendritic cell (BMDC) maturation via IL-10, increased the number of IL-10 producing myeloid-derived suppressor cells, and increased CD4^+^CD25^+^ T cells, thereby effectively suppressing the anti-parasite immune response [47, 49, 63].

Serum IL-10 concentrations of 44.8 pg/mL in the acute stage of *Schistosoma japonicum* infected patients [48] are lower than those in solution concentrations used to validate the biological effects of *IL10RA* variants in vitro. However, IL-10 concentrations in tissue microenvironment are likely higher, and in vitro IL-10 stimulation may not precisely reflect in vivo cytokine–cytokine receptor and producer–consumer stoichiometry as described for other cytokines such as IL-2 [64].

Whereas *S. japonicum* modulates immune responses via the expression of host IL-10, there are viral functional orthologs of cellular IL-10 encoded in the genome of Herpesvirales and *Poxviridae* that can directly modulate host immunity [65]. Due to the geographic distribution and the typically mild disease course, it is unlikely that endemic herpesviruses such as human cytomegalovirus and Epstein-Barr virus drive this selection process of pathogenic *IL10RA* variants via viral IL-10 but historic outbreaks of *Poxviridae* might have been a potential evolutionary selection force. Interestingly, EBV infection is more common in children and adolescents in southern and northern China compared to western countries, indicating differences in complex host–pathogen interaction in these regions [66, 67], and these may involve differences in IL-10 responsiveness.

Surprisingly, we could not find equivalent evidence for balancing natural selection involving the IL-10 or IL-10 receptor genes in geographic regions with endemic *Schistosoma haematobium* and *Schistosoma mansoni*. These differences may reflect distinct urogenital tissue tropism of *S. haematobium* and hepato-intestinal tissue tropism in the cases of *S. mansoni* and *S. japonicum* [68–70]. Differences in the nature of the infection such as in granuloma formation due to distinct modulation of host immune responses through active interference with CXCL8 function by *S. mansoni* but not *S. japonicum* [71], and *S. mansoni*-specific hepatotoxicity [72] may determine alternative selection pressure.

Importantly, the implications of balancing selection of pathogenic *IL10RA* variants in East Asia extend beyond a rare childhood disease. It has the potential to influence our understanding of the immune interaction between IL-10 and parasitic and other infections and could lead to future strategies to targeted prevention and treatment of pathogens. Similar to *Schistosoma japonicum*, the level of IL-10 have a direct impact on protective immunity in *Leishmaniasis major* and *Leishmaniasis tropica* infection models [73, 74]. Genetic IL-10 deficiency as well as transient blockade of IL-10 signaling via anti-IL-10 receptor antibodies resulted in sterile cure of *Leishmaniasis major*, pointing towards a potential therapeutic mechanism [74]. It is tempting to speculate that natural balancing selection of pathogenic *IL10RA* variants constitutes an optimal equilibrium reducing IL-10 signaling in favor of effector pathways that reduce parasite burden without compromising the ability of IL-10 to mediate inflammation control towards commensal intestinal bacteria. This is potentially relevant for hundreds of millions of individuals infected with those IL-10 dependent pathogens and is a strong genetic support to evaluate short term IL-10 balancing therapies. Indeed, targeting the IL-10R not only via antibodies that have a long half-life but aptamers [75], small molecule inhibitors, or bacteria engineered for intestinal IL-10 delivery [76] might allow partial and transient modulation of this pathway.

Compared to Sickle cell disease, a well-studied example of balancing natural selection, the frequencies of disease-causing *IL10RA* alleles are lower. Reasons may be less biological benefit of the heterozygous *IL10RA* variants compared to the Sickle cell disease causing hemoglobin variants that confer selective advantage in the malaria setting and higher mortality of *IL10RA* variants in the homozygous state. In high-income countries, patients with Sickle cell disease have a median survival of 67 years while mortality estimates in sub-Saharan Africa and India, where access to high standard medical care is limited, indicate that 50–90% of patients with Sickle cell disease pass away before 5 years of age [77]. This means comparably higher numbers of patients with Sickle cell disease reach reproductive age compared to biallelic *IL10RA* signaling defects that cause untreated neonatal or infantile mortality.

Although our data strongly suggest that the accumulation of multiple pathogenic *IL10RA* variants in East Asia is not a chance finding, we cannot definitively link those genetic variants to one single infectious disorder. This is due to a lack of granularity of our genetic and infection spatial data, due to the lacking historical perspective (the *IL10RA* variants have arisen as a consequence of historic selection pressure) or might reflect the fact that indeed several pathogens successfully adapted IL-10-dependent strategies in this region. Analysis of large- scale health care utilization datasets in China [78] will allow to apply Mendelian randomization techniques to investigate whether heterozygous IL10RA variants confer improved outcome after certain infections.

## Supplementary Information

Below is the link to the electronic supplementary material.

Supplementary file1 (PNG 239 KB)

Supplementary file2 (PNG 139 KB)

Supplementary file3 (PNG 321 KB)

Supplementary file4 (PNG 195 KB)

Supplementary file5 (PNG 73 KB)

Supplementary file6 (PNG 358 KB)

Supplementary file7 (XLSX 24 KB)

Supplementary file8 (DOCX 80 KB)

Supplementary file9 (XLSX 21 KB)

Supplementary file10 (XLSX 14 KB)

## Data Availability

The data supporting the findings described in this study are available from the corresponding author upon request.

## Code Availability

Computer codes supporting the findings described in this study are available from the corresponding author upon request.
