# Supplementary figures and images for: Pathogenic Interleukin-10 Receptor Alpha Variants in Humans — Balancing Natural Selection and Clinical Implications
Source: J Clin Immunol. 2022 Nov 12;43(2):495–511. doi: 10.1007/s10875-022-01366-7 (PMC9892166; doi:10.1007/s10875-022-01366-7)

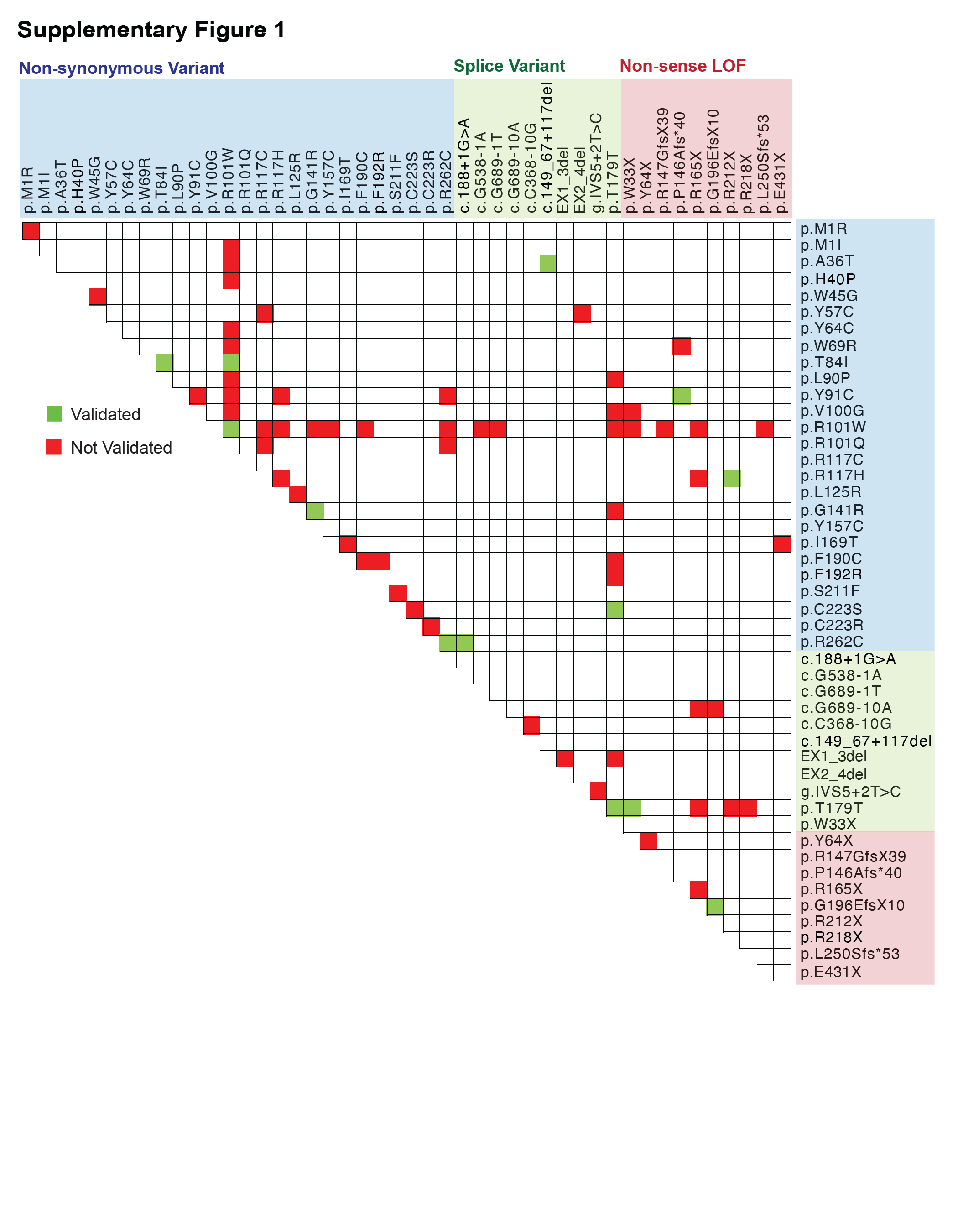

Supplement: Supplementary file 1 — Supplementary file1 (PNG 239 KB) [file 10875_2022_1366_MOESM1_ESM.png]

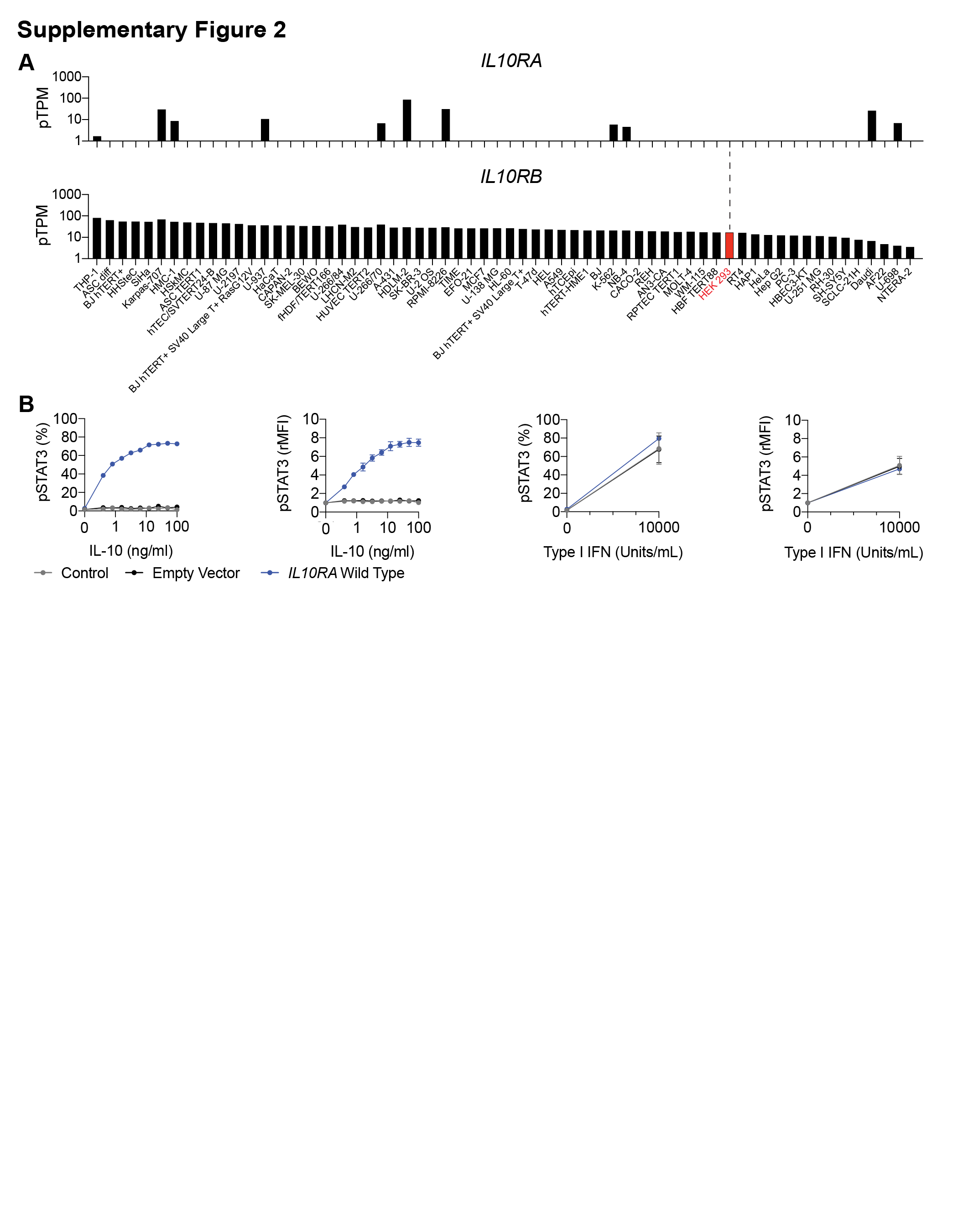

Supplement: Supplementary file 2 — Supplementary file2 (PNG 139 KB) [file 10875_2022_1366_MOESM2_ESM.png]

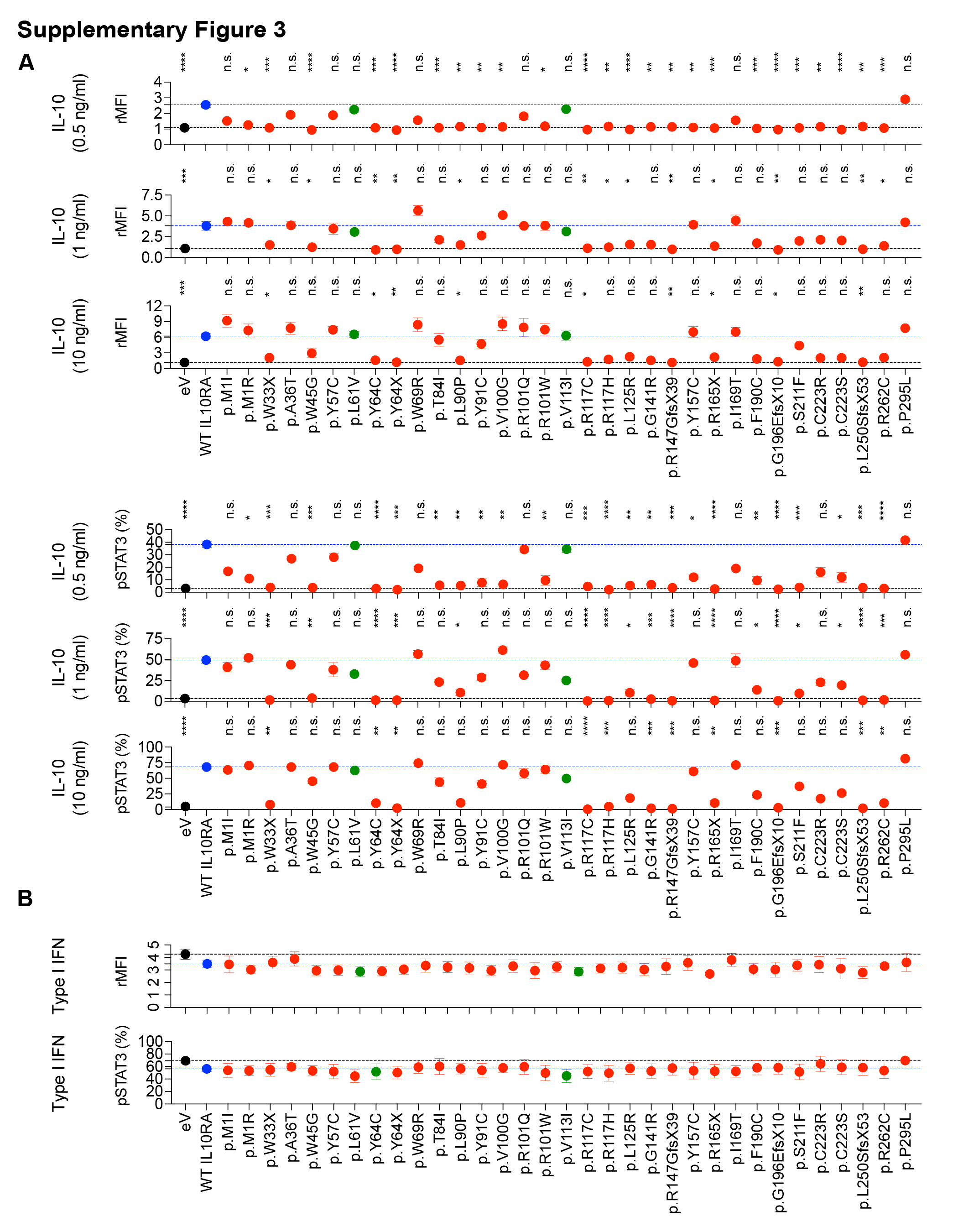

Supplement: Supplementary file 3 — Supplementary file3 (PNG 321 KB) [file 10875_2022_1366_MOESM3_ESM.png]

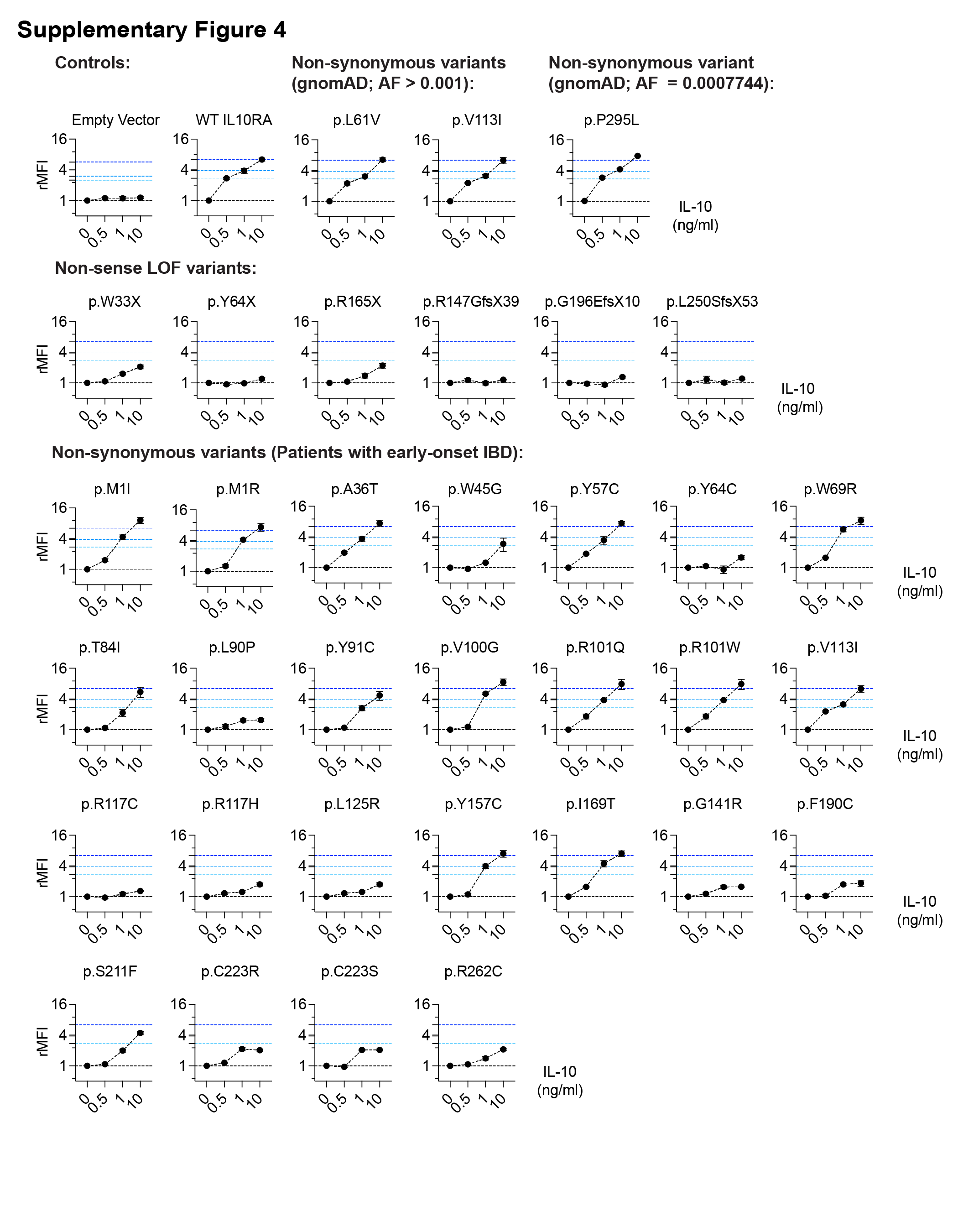

Supplement: Supplementary file 4 — Supplementary file4 (PNG 195 KB) [file 10875_2022_1366_MOESM4_ESM.png]

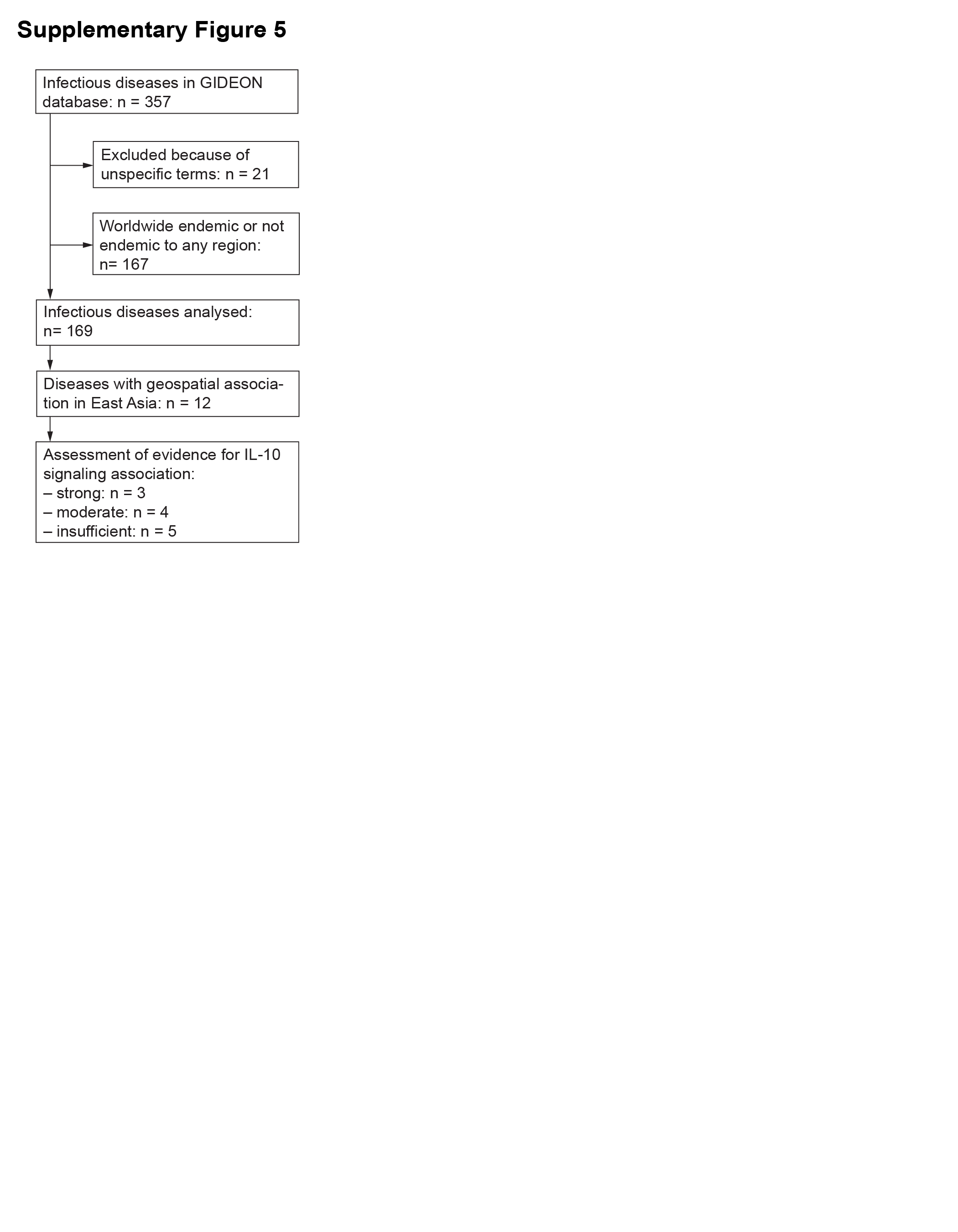

Supplement: Supplementary file 5 — Supplementary file5 (PNG 73 KB) [file 10875_2022_1366_MOESM5_ESM.png]

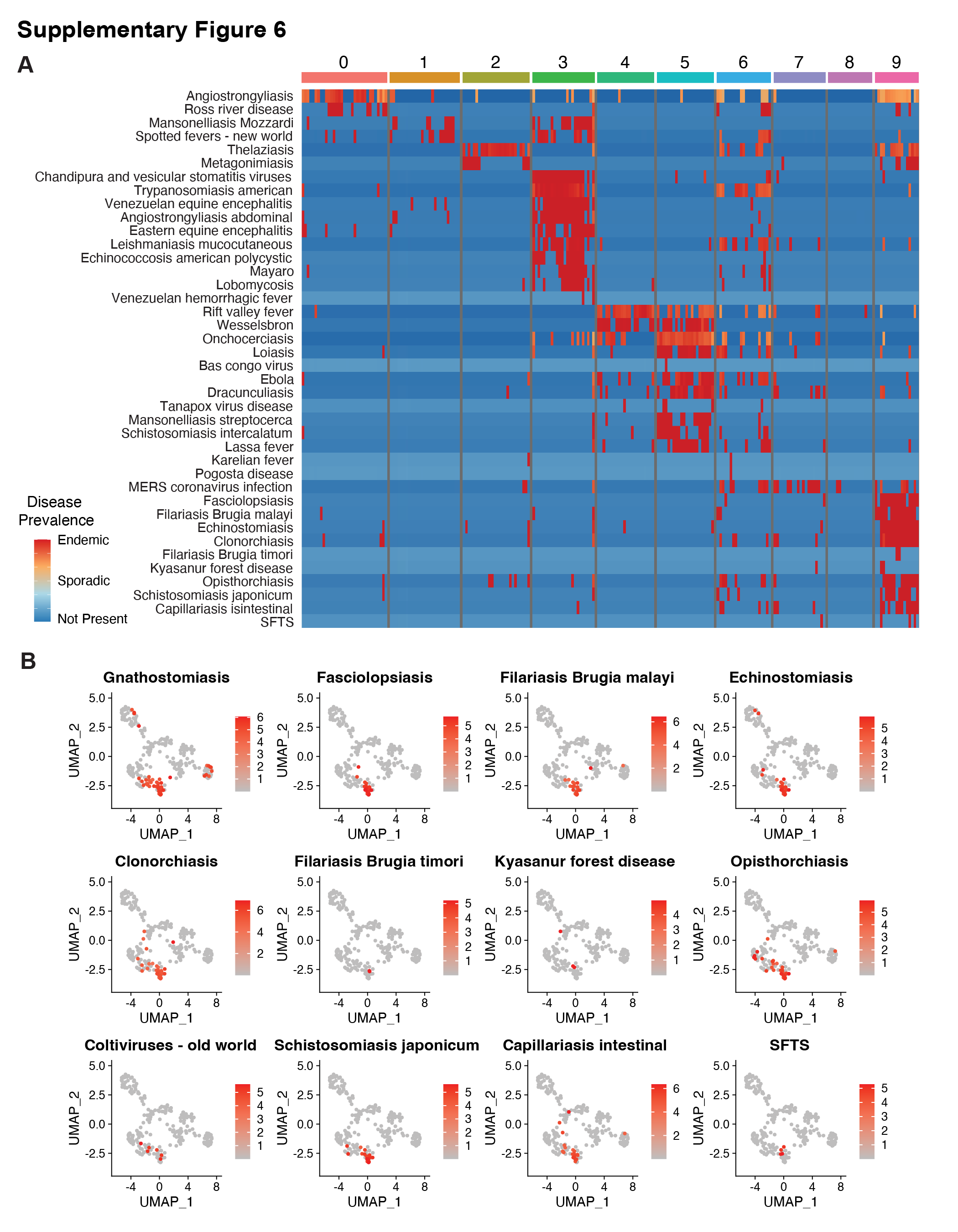

Supplement: Supplementary file 6 — Supplementary file6 (PNG 358 KB) [file 10875_2022_1366_MOESM6_ESM.png]
