# Supplementary material for: Pathogenic Interleukin-10 Receptor Alpha Variants in Humans — Balancing Natural Selection and Clinical Implications
Source: J Clin Immunol. 2022 Nov 12;43(2):495–511. doi: 10.1007/s10875-022-01366-7 (PMC9892166; doi:10.1007/s10875-022-01366-7)
Supplement: Supplementary file 8 — Supplementary file8 (DOCX 80 KB) [file 10875_2022_1366_MOESM8_ESM.docx]

**Table S2**

**Reported cases of VEO-IBD with variants in *IL10*, *IL10RA* and *IL10RB***

| **Gene** |  | ***IL10*** | ***IL10RA*** | ***IL10RB*** |
| --- | --- | --- | --- | --- |
| 2009 | Glocker EO^1^ | 0 | 2 | 2 |
| 2010 | Glocker EO^2^ | 2 | 0 | 0 |
| 2011 | Begue B^3^ | 0 | 1 | 1 |
| 2012 | Mao H^4^ | 0 | 1 | 0 |
| 2012 | Kotlarz D^5^ | 3 | 3 | 7* |
| 2013 | Dinwiddie DL^6^ | 0 | 2 | 0 |
| 2013 | Pigneur B^7^ | 0 | 1 | 3* |
| 2013 | Moran CJ^8^ | 0 | 1 | 0 |
| 2013 | Engelhardt KR^9^ | 0 | 4 | 3 |
| 2014 | Kammermeier J^10^ | 0 | 1 | 0 |
| 2014 | Murugan D^11^ | 0 | 3 | 0 |
| 2014 | Lee CH^12^ | 0 | 1 | 0 |
| 2014 | Shim JO^13^ | 0 | 7 | 0 |
| 2015 | Beser OF^14^ | 0 | 2 | 1 |
| 2015 | Lu D^15^ | 0 | 2 | 0 |
| 2016 | Shouval DS^16^ | 0 | 4 | 2 |
| 2016 | Oh SH^17^ | 0 | 3 | 0 |
| 2016 | Shouval DS^18^ | 0 | 0 | 1 |
| 2016 | Yanagi T^19^ | 0 | 1 | 0 |
| 2016 | Karaca NE^20^ | 0 | 0 | 1 |
| 2016 | Liu LL^21^ | 0 | 5 | 0 |
| 2016 | Xiao Y^22^ | 0 | 3 | 0 |
| 2017 | Petersen BS^23^ | 0 | 1 | 2 |
| 2017 | Veenbergen S^24^ | 0 | 1 | 0 |
| 2017 | McDonald BS^25^ | 0 | 1 | 0 |
| 2017 | Suzuki T^26^ | 0 | 2 | 0 |
| 2017 | Nemati S^27^ | 0 | 1 | 0 |
| 2018 | Zheng C^28^ | 0 | 60 | 1 |
| 2018 | Jung ES^29^ | 0 | 1 | 0 |
| 2018 | Fang YH^30^ | 0 | 4 | 1 |
| 2018 | Ishige T^31^ | 0 | 1 | 0 |
| 2018 | Lu KY^32^ | 0 | 1 | 0 |
| 2019 | Oh SH^33^ | 0 | 1 | 0 |
| 2019 | Teng X^34^ | 0 | 3 | 0 |
| 2019 | Yazdani R^35^ | 0 | 0 | 1 |
| 2019 | Gong YZ^36^ | 0 | 7 | 1 |

*Duplicate cases reported more than once were removed.

Reference

1. Glocker EO, Kotlarz D, Boztug K, et al. Inflammatory bowel disease and mutations affecting the interleukin-10 receptor. N Engl J Med 2009;361:2033-45.

2. Glocker EO, Frede N, Perro M, et al. Infant colitis--it's in the genes. Lancet 2010;376:1272.

3. Begue B, Verdier J, Rieux-Laucat F, et al. Defective IL10 signaling defining a subgroup of patients with inflammatory bowel disease. Am J Gastroenterol 2011;106:1544-55.

4. Mao H, Yang W, Lee PP, et al. Exome sequencing identifies novel compound heterozygous mutations of IL-10 receptor 1 in neonatal-onset Crohn's disease. Genes Immun 2012;13:437-42.

5. Kotlarz D, Beier R, Murugan D, et al. Loss of interleukin-10 signaling and infantile inflammatory bowel disease: implications for diagnosis and therapy. Gastroenterology 2012;143:347-55.

6. Dinwiddie DL, Bracken JM, Bass JA, et al. Molecular diagnosis of infantile onset inflammatory bowel disease by exome sequencing. Genomics 2013;102:442-7.

7. Pigneur B, Escher J, Elawad M, et al. Phenotypic characterization of very early-onset IBD due to mutations in the IL10, IL10 receptor alpha or beta gene: a survey of the Genius Working Group. Inflamm Bowel Dis 2013;19:2820-8.

8. Moran CJ, Walters TD, Guo CH, et al. IL-10R polymorphisms are associated with very-early-onset ulcerative colitis. Inflamm Bowel Dis 2013;19:115-23.

9. Engelhardt KR, Shah N, Faizura-Yeop I, et al. Clinical outcome in IL-10- and IL-10 receptor-deficient patients with or without hematopoietic stem cell transplantation. J Allergy Clin Immunol 2013;131:825-30.

10. Kammermeier J, Drury S, James CT, et al. Targeted gene panel sequencing in children with very early onset inflammatory bowel disease--evaluation and prospective analysis. J Med Genet 2014;51:748-55.

11. Murugan D, Albert MH, Langemeier J, et al. Very early onset inflammatory bowel disease associated with aberrant trafficking of IL-10R1 and cure by T cell replete haploidentical bone marrow transplantation. J Clin Immunol 2014;34:331-9.

12. Lee CH, Hsu P, Nanan B, et al. Novel de novo mutations of the interleukin-10 receptor gene lead to infantile onset inflammatory bowel disease. J Crohns Colitis 2014;8:1551-6.

13. Shim JO, Seo JK. Very early-onset inflammatory bowel disease (IBD) in infancy is a different disease entity from adult-onset IBD; one form of interleukin-10 receptor mutations. J Hum Genet 2014;59:337-41.

14. Beser OF, Conde CD, Serwas NK, et al. Clinical features of interleukin 10 receptor gene mutations in children with very early-onset inflammatory bowel disease. J Pediatr Gastroenterol Nutr 2015;60:332-8.

15. Lu D, Xu Y, Chen Y, et al. [Interleukin-10 receptor mutations in children with neonatal onset inflammatory bowel disease: genetic diagnosis and pathogenesis]. Zhonghua Er Ke Za Zhi 2015;53:348-54.

16. Shouval DS, Biswas A, Kang YH, et al. Interleukin 1beta Mediates Intestinal Inflammation in Mice and Patients With Interleukin 10 Receptor Deficiency. Gastroenterology 2016;151:1100-1104.

17. Oh SH, Baek J, Liany H, et al. A Synonymous Variant in IL10RA Affects RNA Splicing in Paediatric Patients with Refractory Inflammatory Bowel Disease. J Crohns Colitis 2016;10:1366-1371.

18. Shouval DS, Ebens CL, Murchie R, et al. Large B-Cell Lymphoma in an Adolescent Patient With Interleukin-10 Receptor Deficiency and History of Infantile Inflammatory Bowel Disease. J Pediatr Gastroenterol Nutr 2016;63:e15-7.

19. Yanagi T, Mizuochi T, Takaki Y, et al. Novel exonic mutation inducing aberrant splicing in the IL10RA gene and resulting in infantile-onset inflammatory bowel disease: a case report. BMC Gastroenterol 2016;16:10.

20. Karaca NE, Aksu G, Ulusoy E, et al. Early Diagnosis and Hematopoietic Stem Cell Transplantation for IL10R Deficiency Leading to Very Early-Onset Inflammatory Bowel Disease Are Essential in Familial Cases. Case Reports Immunol 2016;2016:5459029.

21. Fumagalli M, Pozzoli U, Cagliani R, et al. Parasites represent a major selective force for interleukin genes and shape the genetic predisposition to autoimmune conditions. J Exp Med 2009;206:1395-408.

22. Xiao Y, Wang XQ, Yu Y, et al. Comprehensive mutation screening for 10 genes in Chinese patients suffering very early onset inflammatory bowel disease. World J Gastroenterol 2016;22:5578-88.

23. Petersen BS, August D, Abt R, et al. Targeted Gene Panel Sequencing for Early-onset Inflammatory Bowel Disease and Chronic Diarrhea. Inflamm Bowel Dis 2017;23:2109-2120.

24. Veenbergen S, van Leeuwen MA, Driessen GJ, et al. Development and Function of Immune Cells in an Adolescent Patient With a Deficiency in the Interleukin-10 Receptor. J Pediatr Gastroenterol Nutr 2017;65:e5-e15.

25. McDonald BS, Narayanan S, Elawad M, et al. Interleukin-10 receptor mutation presenting with severe nappy ulceration and infantile inflammatory bowel disease. Clin Exp Dermatol 2017;42:771-773.

26. Suzuki T, Sasahara Y, Kikuchi A, et al. Targeted Sequencing and Immunological Analysis Reveal the Involvement of Primary Immunodeficiency Genes in Pediatric IBD: a Japanese Multicenter Study. J Clin Immunol 2017;37:67-79.

27. Nemati S, Teimourian S, Tabrizi M, et al. Very early onset inflammatory bowel disease: Investigation of the IL-10 signaling pathway in Iranian children. Eur J Med Genet 2017;60:643-649.

28. Zheng C, Huang Y, Hu W, et al. Phenotypic Characterization of Very Early-Onset Inflammatory Bowel Disease with Interleukin-10 Signaling Deficiency: Based on a Large Cohort Study. Inflamm Bowel Dis 2019;25:756-766.

29. Jung ES, Petersen BS, Mayr G, et al. Compound heterozygous mutations in IL10RA combined with a complement factor properdin mutation in infantile-onset inflammatory bowel disease. Eur J Gastroenterol Hepatol 2018;30:1491-1496.

30. Fang YH, Luo YY, Yu JD, et al. Phenotypic and genotypic characterization of inflammatory bowel disease in children under six years of age in China. World J Gastroenterol 2018;24:1035-1045.

31. Ishige T, Igarashi Y, Hatori R, et al. IL-10RA Mutation as a Risk Factor of Severe Influenza-Associated Encephalopathy: A Case Report. Pediatrics 2018;141.

32. Huang Z, Peng K, Li X, et al. Mutations in Interleukin-10 Receptor and Clinical Phenotypes in Patients with Very Early Onset Inflammatory Bowel Disease: A Chinese VEO-IBD Collaboration Group Survey. Inflamm Bowel Dis 2017;23:578-590.

33. Oh SH, Sung YH, Kim I, et al. Novel Compound Heterozygote Mutation in IL10RA in a Patient With Very Early-Onset Inflammatory Bowel Disease. Inflamm Bowel Dis 2019;25:498-509.

34. Teng X, Xu L, Sun M, et al. Phenotypic characteristics and clinical manifestations of inflammatory bowel disease in infants and children under 2 years of age in Liaoning Province, China: five of six infants with IL-10R mutations. Paediatr Int Child Health 2019;39:59-64.

35. Yazdani R, Moazzami B, Madani SP, et al. Candidiasis associated with very early onset inflammatory bowel disease: First IL10RB deficient case from the National Iranian Registry and review of the literature. Clin Immunol 2019;205:35-42.

36. Gong YZ, Ning HJ, Ma X, et al. [Clinical and genotypic characteristics of infantile inflammatory bowel disease]. Zhonghua Er Ke Za Zhi 2019;57:520-525.
